# INSaFLU-TELEVIR: an open web-based bioinformatics suite for viral metagenomic detection and routine genomic surveillance

**DOI:** 10.1186/s13073-024-01334-3

**Published:** 2024-04-25

**Authors:** João Dourado Santos, Daniel Sobral, Miguel Pinheiro, Joana Isidro, Carlijn Bogaardt, Miguel Pinto, Rodrigo Eusébio, André Santos, Rafael Mamede, Daniel L. Horton, João Paulo Gomes, TELEVIR Consortium, Laurent Bigarré, Jovita Fernández-Pinero, Ricardo J. Pais, Maurilia Marcacci, Ana Moreno, Tobias Lilja, Øivind Øines, Artur Rzeżutka, Elisabeth Mathijs, Steven Van Borm, Morten Rasmussen, Katja Spiess, Vítor Borges

**Affiliations:** 1 Genomics and Bioinformatics Unit, Department of Infectious Diseases, National Institute of Health Doutor Ricardo Jorge (INSA), Lisbon, Portugal; 2 Institute of Biomedicine-iBiMED, Department of Medical Sciences, University of Aveiro, Aveiro, Portugal; 3 Department of Comparative Biomedical Sciences, School of Veterinary Medicine, University of Surrey, Surrey, UK; 4 Faculdade de Medicina, Instituto de Microbiologia, Instituto de Medicina Molecular, Universidade de Lisboa, Lisbon, Portugal; 5 Veterinary and Animal Research Centre (CECAV), Faculty of Veterinary Medicine, Lusófona University, Lisbon, Portugal

## Abstract

**Background:**

Implementation of clinical metagenomics and pathogen genomic surveillance can be particularly challenging due to the lack of bioinformatics tools and/or expertise. In order to face this challenge, we have previously developed INSaFLU, a free web-based bioinformatics platform for virus next-generation sequencing data analysis. Here, we considerably expanded its genomic surveillance component and developed a new module (TELEVIR) for metagenomic virus identification.

**Results:**

The routine genomic surveillance component was strengthened with new workflows and functionalities, including (i) a reference-based genome assembly pipeline for Oxford Nanopore technologies (ONT) data; (ii) automated SARS-CoV-2 lineage classification; (iii) Nextclade analysis; (iv) Nextstrain phylogeographic and temporal analysis (SARS-CoV-2, human and avian influenza, monkeypox, respiratory syncytial virus (RSV A/B), as well as a “generic” build for other viruses); and (v) *algn2pheno* for screening mutations of interest. Both INSaFLU pipelines for reference-based consensus generation (Illumina and ONT) were benchmarked against commonly used command line bioinformatics workflows for SARS-CoV-2, and an INSaFLU snakemake version was released. In parallel, a new module (TELEVIR) for virus detection was developed, after extensive benchmarking of state-of-the-art metagenomics software and following up-to-date recommendations and practices in the field. TELEVIR allows running complex workflows, covering several combinations of steps (e.g., with/without viral enrichment or host depletion), classification software (e.g., Kaiju, Kraken2, Centrifuge, FastViromeExplorer), and databases (RefSeq viral genome, Virosaurus, etc.), while culminating in user- and diagnosis-oriented reports. Finally, to potentiate real-time virus detection during ONT runs, we developed *findONTime*, a tool aimed at reducing costs and the time between sample reception and diagnosis.

**Conclusions:**

The accessibility, versatility, and functionality of INSaFLU-TELEVIR are expected to supply public and animal health laboratories and researchers with a user-oriented and pan-viral bioinformatics framework that promotes a strengthened and timely viral metagenomic detection and routine genomics surveillance. INSaFLU-TELEVIR is compatible with Illumina, Ion Torrent, and ONT data and is freely available at https://insaflu.insa.pt/ (online tool) and https://github.com/INSaFLU (code).

**Supplementary Information:**

The online version contains supplementary material available at 10.1186/s13073-024-01334-3.

## Background

Infectious diseases pose a constant and serious threat to human and animal populations. As such, modern surveillance systems should be able to detect and track the emergence and circulation of (new or variants of known) pathogens, as well as monitor their phenotypic and epidemiological relevant features. With the advances in next-generation sequencing (NGS), whole-genome sequencing (WGS) rapidly became the method of choice for a fine resolution of pathogens’ genetic relatedness and exploration of evolutionary and genomics features of interest, such as antimicrobial resistance and virulence traits [1–6]. In the virology field, the COVID-19 pandemic and other recent international public health threats (e.g., the multi-country mpox outbreak and the A/H5N1 avian influenza global spread) [7, 8] have particularly triggered this rampant technological transition, consolidating virus genome sequencing as the gold standard tool for outbreak detection and tracking, with benefits for guiding diagnostics, prophylaxis, and research [4–6, 9–11]. In parallel, the application of NGS for clinical microbiology, through non-targeted metagenomics, is another field in rapid expansion and poised to complement traditional diagnostic methods. This paradigm shift has been well reflected in an increasing awareness of the added value of genomic surveillance by decision makers (leading to increasing funding initiatives) [12], but especially in the great efforts of the scientific community to develop and share laboratory protocols and bioinformatics tools for sequence data generation and analysis, to reinforce (or establish new) intra- and inter-country laboratory networks, and promote training initiatives [13–15]. Nonetheless, there are still huge discrepancies between countries, sectors, and/or laboratories in the implementation of viral metagenomic diagnostics and routine genomic surveillance, often due to limited availability of (i) computational infrastructure and/or specialized personnel to process and interpret NGS data; (ii) automated, standardized and scalable bioinformatics workflows for metagenomics-based pathogen detection and routine genomic surveillance; and/or (iii) tools for systematic and comprehensive integration of genomics data with clinical, demographic and epidemiological data [16, 17]. In addition, with the capability for (near) real-time analyses during sequencing runs, enabled by technologies like Oxford Nanopore Technologies (ONT), there is a demand for novel bioinformatics tools that efficiently enhance this feature, towards reduced turn-around times and sequencing costs. Particularly crucial in clinical diagnostics is also the development of innovative bioinformatics for both analysis and reporting that can tackle the known issue of high false positive rates associated with taxonomic classification tools [18]. In order to catch up with this technological revolution, and aiming to increase the capacity of laboratories with fewer resources, we have previously developed INSaFLU [19], an open and innovative web-based bioinformatics platform for virus NGS data analysis. On behalf of the One Health European Joint Programme (OHEJP) TELEVIR (https://onehealthejp.eu/projects/emerging-threats/jrp-tele-vir) [20] project, along with the development of wet-lab protocols [21], and also as a response to public health threats (namely, the COVID-19 pandemic and the multi-country mpox outbreak), we expanded its genomic surveillance component and developed a brand new module (TELEVIR) for viral metagenomic identification. In this study, we present the upgraded INSaFLU-TELEVIR platform (https://insaflu.insa.pt/) [22], a free, versatile, and user-oriented “start-to-end” pan-viral bioinformatics framework aiming at facilitating or strengthening the laboratories capacity building in genomic epidemiology and public and animal health bioinformatics towards an enhanced and global surveillance of viral threats.

## Implementation

### Viral metagenomic detection

#### Workflow overview and rationale

**Fig. 1:**
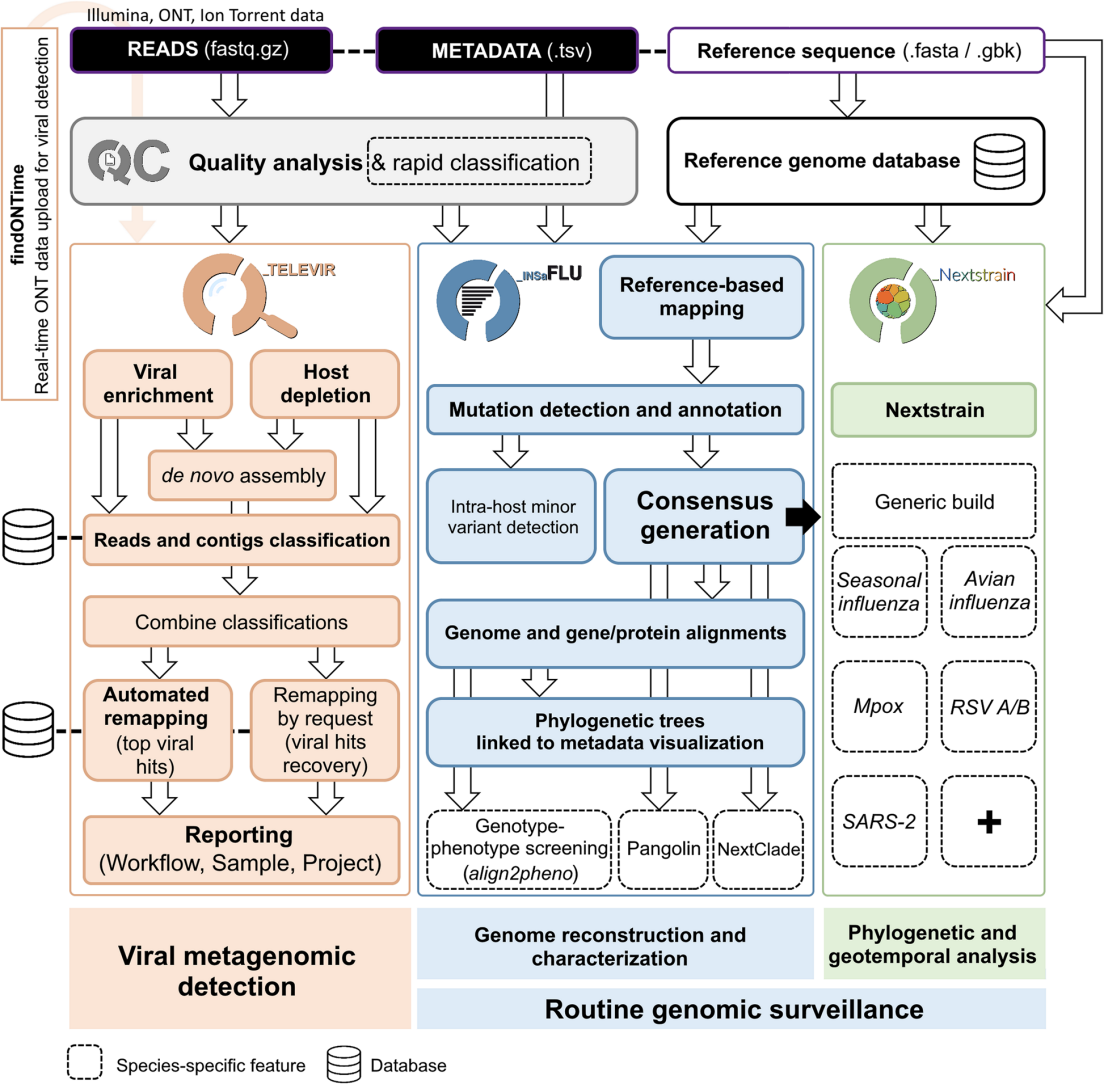
Architecture of the INSaFLU-TELEVIR platform, summarizing the implemented analytical modules for viral metagenomic detection (TELEVIR) and routine genomic surveillance (INSaFLU and NextStrain)

**Fig. 2:**
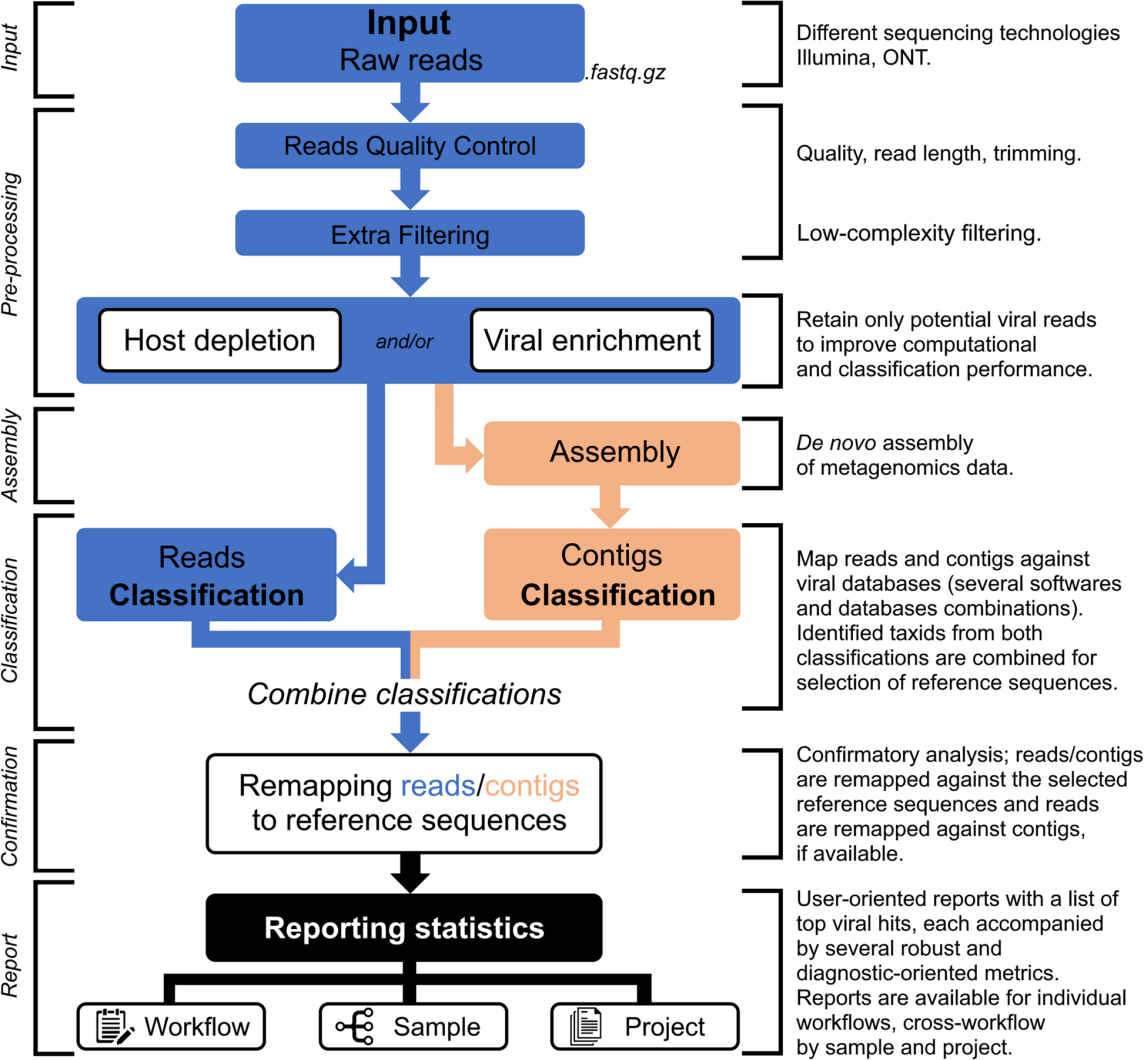
Simplified illustration of the main steps of the modular INSaFLU-TELEVIR bioinformatics pipeline for metagenomics virus detection (TELEVIR). Documentation for each step is provided at the website (https://insaflu.insa.pt)

One of the main developments since INSaFLU’s first release [19] focused on upgrading the platform for automated metagenomic virus identification, in order to support both human and veterinary clinical practice and disease outbreak investigations. After reviewing the current state-of-the-art field of bioinformatics pipelines for metagenomic virus diagnostics [18, 23–28] and consulting the TELEVIR consortium (Public Health and Veterinary institutes across all Europe), a modular pipeline was designed and developed, incorporating the key steps of NGS metagenomics taxonomic classification and reporting (Figs. 1 and 2), namely: read quality control, viral enrichment/host depletion, de novo assembly, reads/contigs taxonomic classification, and confirmatory reference-based remapping and reporting. The choice of the internal components of the implemented workflows (software, default parameters, etc.) resulted from an extensive benchmarking (next section). Details of TELEVIR resources, benchmarking, and implementation are detailed in Additional file 1. In summary, the input/output flow and main functionalities behind the main TELEVIR steps are as follows:

- *Read quality analysis and improvement:* This step takes the input single- or paired-end reads (fastq.gz format; Illumina, Ion Torrent, or ONT) and produces quality processed reads, as well as quality control reports for each file, before and after this step. This step is performed automatically following sample upload and thus overlaps the two components (virus detection and genomic surveillance) of the INSaFLU-TELEVIR platform. Quality filtering and trimming of Illumina reads is performed as described in Borges et al. (2018) [19], and treatment of ONT data is described below. An optional, extra filtering layer that targets low-complexity reads is available as part of the TELEVIR pipeline using the software PRINSEQ [29]. Parameters are modifiable by the user.
- *Viral enrichment*: This step retains potential viral reads based on a rapid and permissive classification of the reads against a viral sequence database. This step is performed directly over raw reads (if QC was turned OFF) or quality processed reads (if QC was turned ON).
- *Host depletion:* This step removes potential host reads based on reference-based mapping against host genome sequence(s). Mapped reads are treated as potential host reads and removed. This step will act on virus-enriched sequences, unless the viral enrichment step was turned OFF, in which case host depletion will be directly performed over raw/quality processed reads. Several host sequences are provided as default.
- De novo* assembly*: This step performs de novo assembly using reads retained after the “Viral enrichment” and/or “Host depletion” steps. If the latter steps were turned OFF, assembly will be directly performed using raw / processed reads. Assembled contigs are automatically filtered for a minimum sequence length.
- *Identification of viral sequences*: This step screens reads and contigs against viral sequence databases, generating an intermediate read and/or contig classification report: a list of viral hits (taxonomic identifiers (TAXID) and representative accession identifiers (ACCID)) potentially present in the sample. TAXIDs bearing the keyword “phage” in their scientific name are filtered out.
- *Selection of viral TAXID and representative genome sequences for confirmatory analysis*: In this step, the previously identified viral hits (TAXID) are selected for confirmatory mapping against reference viral genome(s) present in the available databases. Viral TAXIDs are selected, up to a maximum number of hits, under the following order: (i) viral hits corresponding to phages are removed from classification report; (ii) TAXIDs present in both intermediate classification reports (reads and contigs) are selected; (iii) additional TAXIDs are selected across the read classification report and contigs classification report by number of hits, in decreasing order, and total length of matching sequences, when available, until reaching the defined maximum number of hits to be selected (this number is to be user-defined). Finally, TAXIDs are queried against available databases for associated ACCIDs.
- *Remapping of the viral sequences against selected reference genomes*: This step map reads and contigs against representative genome sequences (ACCIDs) of the selected viral TAXIDs collected in the previous step. Reads are also mapped against the set of contigs classified for each TAXID. Of note, TAXIDs that were not automatically selected for this confirmatory remapping step (but that were present in the intermediate reads and/or contigs classification reports) can still be user-selected for mapping at any time. An optional, extra layer of “mapping stringency” was added to this step to minimize false positive hits, allowing users to set a maximum sum of the mismatch qualities before marking a read unmapped and a maximum fraction of nucleotide mismatches allowed before soft clipping from ends. This additional layer is optional and disabled by default.
- *Reporting:* The workflow culminates in user-oriented reports on a list of the top suspected viruses (detailed in the “Usage” section), each accompanied by several diagnostic-oriented metrics, statistics, and visualizations, provided as (interactive) tables (intermediate and final reports), graphs (e.g., coverage plots, Integrative Genomics Viewer visualization, Assembly to reference dotplots) and multiple downloadable output files (e.g., list of the software parameters, reads/contigs classification reports, mapped reads/contigs identified per each virus; reference sequences, etc.). To further help the user in assessing the validity of the reported hits in a given sample, viral references are grouped by mapping overlap, as measured by the number of shared mapped reads. This grouping is capable of placing together true positive hits with their corresponding cross-mapped potential false positives, allowing for the easy identification of the latter. Grouping parameters are modifiable in the Reporting section of the TELEVIR Settings Menu for both sequencing technologies.

In the context of metagenomics in clinical virology, cross-mapping of reads across several host and pathogen reference sequences is very common, resulting in a high false positive rate [30–32]. The TELEVIR workflow provides a light-weight but robust approach, in classification and interpretation, to false positives. Firstly, it follows suggestions expressed in the literature to first filter out reads enriched with low complexity regions (e.g., homopolymeric tracts or short-repeats), as well as unwanted material (host or non-viral “contaminants”) through host depletion and/or viral enrichment [23, 28, 33]. These steps aim at decreasing background noise and increasing the performance [28, 34, 35] and the speed of both read classifiers and assemblers. In turn, besides the reads classification, the workflow takes advantage of the increased precision of contig classification [36], which provides an additional, robust metric with which to assess the validity of the final results. This pipeline further innovates in tackling false positives by introducing a final confirmatory analysis that comprises automatic reference selection, remapping (including optional “mapping stringency” settings), hits grouping, and warning flagging. By normalizing the classification reports and outputs for comparison, the interactive reports provide a uniform basis on which to confirm classifications and weed out false positives.

#### TELEVIR benchmarking

In order to finely explore the best approaches to be implemented in the TELEVIR toolbox, we benchmarked several workflows. For this, we tested combinations of the key modules that comprise the overall virus identification pipeline: Viral Enrichment, Assembly, and Classification (of reads and contigs). Specifically, we tested software (such as Centrifuge, Diamond, Kaiju, Kraken2, KrakenUniq, and BLAST) (Table S1) and databases (such as NCBI, RVDB, UniProt, and Virosaurus) (Table S2) commonly used in virological diagnostic laboratories for clinical metagenomics, as well as some more recent but promising alternative classifiers (deSAMBA, FastViromeExplorer, Clark) (see mode details in Additional file 1—Resources). In some instances, we also evaluated software performance by varying argument values (Table S3). We further benchmarked the sorting algorithm used to rank candidate reference hits based on the results of the Read and Contig classification steps. For ONT data, we ran 117 combinations (i.e., different software, reference databases, and/or parameters) on 20 samples (a total of 2340 runs). For Illumina data, we ran 108 combinations on 24 samples (2592 total runs). The reads used in the benchmark (Table S4) covered a wide range of viruses (including influenza and SARS-CoV-2, but also bluetongue and epizootic hemorrhagic disease virus, among others) and hosts (including human, various ungulates, and one culicoides specimen), and include a dataset of clinical samples from patients with encephalitis or viral respiratory infections, previously used to benchmark software for metagenomic virus diagnostics [26].

The design, methodological details and results of this extensive benchmark are described in Additional file 1 (covering Tables S1–S7 and Fig. S1–S6). In summary, we found that combining the information from contig and read classification in order to rank metagenomics candidate hits is indeed preferable than depending on a single classification source (Fig. S1). Regarding software selection: at the Viral Enrichment step, Kraken2, and Centrifuge performed the best for Illumina and ONT technologies, respectively, based on precision (Fig. S3A–B); at the Contig classification step, we found that Nucleotide BLAST resulted in the highest number of successfully mapped contigs (Fig. S3C–D); At the Read Classification step, we found significant differences in precision between several softwares for ONT, but not for Illumina (Fig. S3E–F). In the end, our choice of software (Table S8) reflected a trade-off between benchmark results at the module level (Fig. S3) and in combination (Fig. S4), providing the user with adequate cross-validation, and the constraints of implementing new software on an existing platform (see Additional file 1—Section 4.1). Finally, a simulation study shows that the confirmatory mapping step is robust in capturing divergent sequences (Fig. S5). The implications and limitations of this method of confirmation are discussed in Section 3.4.5 of Additional file 1.

#### findONTime, a complementary tool to enable real-time metagenomics virus detection

When performing hypothesis-free viral diagnosis by sequencing complex biological samples, the proportion of the virus in a sample is unknown. As such, the amount of sequencing data, and, consequently, the run length needed to accurately detect a virus cannot be predicted a priori. These result in sequencing runs often being allowed to run overnight, at the expense of the material and, in the context of diagnostics, the potential detriment of patient or animal status. Inspired by existing examples in the field for real-time ONT targeted mapping and overview of genome coverage (e.g., RAMPART; https://artic.network/rampart) [37], we envisaged a tool for continuous ONT run monitoring in the context of viral metagenomics that would allow users to cut short a sequencing run when sufficient pathogen sequence evidence has been gathered. As such, we developed findONTime (https://github.com/INSaFLU/findONTime) [38], a command-line tool complementary to the INSaFLU-TELEVIR platform that potentiates a time and cost-effective real-time viral metagenomic detection. findONTime is a multi-threaded python package that runs concurrently with MinION sequencing to (i) monitor the demultiplexed FASTQ files (gzipped or not) that are being generated in real-time for each sample (the sequencing run should have the barcoding option ON); (ii) merge the same-sample files (at user-defined time intervals), downsize them (on demand) and prepare a metadata table (according to the INSaFLU-TELEVIR template); and, if requested, (iii) upload these files (ONT reads and metadata) to the INSaFLU-TELEVIR platform (local server via SSH or directly through docker, depending on a user-provided configuration file); and (iv) launch the metagenomics virus detection analysis using the TELEVIR module. findONTime (https://github.com/INSaFLU/findONTime) is implemented in python 3.9 and is pip-installable (https://pypi.org/project/findontime/) [38].

### Routine genomic surveillance

#### Reference-based genome assembly

With the recent advances in third-generation sequencing technologies (ONT) and their wider access through more portable and affordable equipments (MinION), it became necessary to deploy a reference-based genome assembly pipeline for ONT data analysis in the INSaFLU-TELEVIR platform, in addition to the existing workflow for Illumina and Ion Torrent data [19]. In order to keep the same dashboard usability across technologies (see Usage section), the implemented ONT workflow followed the same pipeline architecture (from raw reads to quality analysis, reference-based generation/curation of consensus sequences, and mutation detection) and input/output flow and formats [19], but relying on open-source software specifically adapted to the characteristics of ONT data. First, sequencing technology (ONT or Illumina/Ion Torrent) is automatically inferred from the distribution of read lengths, upon read upload. Samples classified as ONT are QC filtered using NanoFilt [39], and statistics and reporting are generated using NanoStat [39] and RabbitQC [40]. Default parameters for NanoFilt, regarding average read quality, minimum/maximum read length, and start/end trimming size (Table S8), were selected to provide a trade-off between quality and read length, but are open to user configuration to fit to sample characteristics and the upstream experimental conditions, etc. Post-QC reads are then processed by medaka (https://github.com/nanoporetech/medaka) [41] using “consensus” and “variant” modes to generate raw consensus sequences (FASTA) and mutation lists (VCF), respectively. After a calculation of the depth of coverage per site, mutations present in the raw VCF files are filtered out based on user-configurable criteria: (i) minimum depth of coverage per site (--mincov) (default: 30) and (ii) minimum proportion for variant evidence (--minfrac) (default: 0.8). Intermediate consensus sequences are then generated using bcftools [42] based on the VCF file containing the validated mutations. The last step of consensus sequence curation involves the automatic placement of undefined nucleotides (“N”) in (i) low coverage regions (i.e., regions with coverage below --mincov), using “msa_masker (https://github.com/rfm-targa/BioinfUtils/blob/master/FASTA/msa_masker.py) [43]; (ii) mutations with frequencies between 50% and the user defined “--minfrac”; and (iii) regions (or sites) selected to be masked by the user (e.g., regions falling outside the amplicon schema). Steps (i) and (iii) of consensus curation were also incorporated in the existing Illumina/Ion Torrent workflow [19], which is also similar in all subsequent steps of mutation annotation (using snpEff) [44], alignment (using MAUVE and MAFFT) [45, 46], and rapid phylogenetics (using FastTree) [47], as previously described [19].

#### INSaFLU benchmarking

The INSaFLU reference-based genome assembly pipeline for Illumina data analysis was previously benchmarked for influenza virus [19] using the IRMA pipeline [48] for comparison. In the present study, we performed additional benchmarking for SARS-CoV-2, comparing INSaFLU with a commonly used command-line bioinformatics workflow (https://github.com/andersen-lab/HCoV-19-Genomics) [49], involving BWA for reads mapping [50] and iVar (https://github.com/andersen-lab/ivar; https://andersen-lab.github.io/ivar/html/manualpage.html) [51] for QC and consensus generation [52]. The newly implemented INSaFLU pipeline for ONT data was also benchmarked against the widely used ARTIC SARS-CoV-2 pipeline (https://github.com/artic-network/fieldbioinformatics/) [53]. The methodological details and results of the two benchmarks are described in Additional file 2. In summary, for Illumina, the comparative analysis of the obtained consensus sequences using INSaFLU versus BWA/iVar workflow demonstrated similar performance by both pipelines, but underlined the expected added value of incorporating an extra step of targeted primer clipping from the BAM file, as implemented in iVar (Additional file 2). In this context, the specific iVar primer clipping step (including primer trimming from aligned reads, as well as removal of aligned reads containing minor variants matching primer sequence but differing from the consensus sequence) was incorporated in both Illumina and ONT pipelines. The primer scheme (used for amplification) is selected in the dashboard by the user upon Project creation and before adding samples. The final benchmark results using the upgraded INSaFLU workflows confirmed that the INSaFLU consensus generation performs similarly to widely used Illumina and ONT pipelines (detailed discussion in Additional file 2).

#### Additional implementation of surveillance-oriented functionalities

In parallel to the refinement of the reference-based genome assembly pipelines, we integrated other important surveillance-oriented (often virus-specific) functionalities and features into the platform. The “References” default database (open to all users) was continuously enriched with genome sequences relevant for surveillance of viruses other than influenza, namely SARS-CoV-2, RSV, and MPXV. The step of rapid virus identification/classification upon reads upload, as originally described [19], was also strengthened by accommodating ONT data, through draft assembly using Raven [54], and by upgrading the database of genetic markers to rapidly identify the presence of human betacoronavirus (namely, HCoV-OC43, HCoV-HKU1, MERS, SARS, and SARS-CoV-2), RSV A and B, as well as MPXV. Pangolin (https://github.com/cov-lineages/pangolin) [55, 56] was incorporated for SARS-CoV-2 Pango lineage classification (default settings, UshER mode [57]), with pango software and databases automatically updated on a daily basis to provide up-to-date (re-)classification of new and old sequences. As a complement, direct hyperlinks to Nextclade (https://clades.nextstrain.org/) [58] are automatically provided for rapid and flexible clade/lineage classification and quality analysis of SARS-CoV-2, seasonal influenza, MPXV, and RSV consensus sequences (INSaFLU consensus sequences are directly analyzed at client side on the browser). Other main improvements of the surveillance component involved the incorporation of Nextstrain (https://nextstrain.org/) [59, 60], and the development and integration of *algn2pheno* (https://github.com/insapathogenomics/algn2pheno) [61], as described in the next sections.

#### Integration of Nextstrain phylogeographic and temporal analysis

Nextstrain (https://nextstrain.org/) [59, 60] relies on reproducible and open-sourced pathogen-specific workflows for genomic data curation, analysis, and visualization of integrated phylogeographical and temporal data, towards a real-time tracking of pathogen evolution. Hence, we strengthened the INSaFLU-TELEVIR surveillance component by integrating Nextstrain phylogeographic and temporal analysis of several viruses, namely SARS-CoV-2, seasonal and avian influenza, MPXV and RSV. We performed minor adjustments to the original Nextstrain workflows in order to (i) change the input source so that the implemented workflow incorporates user-provided sequences (via INSaFLU or by direct user upload) instead of fetching from databases; (ii) relax some of the sequence filtering (more important when fetching from external databases) to maximize the number of input sequences included in the final tree (most often consensus from INSaFLU projects that already passed user-provided quality filters); and (iii) reduce input/output complexity, by removing some pathogen-specific inferences that require metadata that may not always be available (e.g., clinical onset date). For example, in the specific case of seasonal influenza, we removed fitness inferences and allowed more ambiguity and divergence among input sequences. Moreover, users may want to analyze organisms for which there is no specific Nextstrain build available. To cover these cases, we included a generic build (with or without temporal data) that performs alignment and tree-building using augur [62], either with the user-provided reference as root (when temporal data is not provided), or inferring the root and molecular clock from user-provided temporal sample metadata. The generic builds and the INSaFLU-adapted species-specific Nextstrain workflows are kept in a separate repository available at https://github.com/INSaFLU/nextstrain_builds [63]. We regularly update our workflows with changes to the repositories of the original workflows, namely, e.g., with information regarding clades.

#### *algn2pheno*—screening of potential genotype–phenotype associations

In the course of the evolution of any given pathogen, multiple mutations and combinations of mutations are continuously arising. Although many/most of these mutations usually have no effect or relevance, sometimes it is possible to associate some mutations with specific phenotypes or characteristics (such as antiviral resistance, resistance to neutralizing antibodies, enhanced affinity to host-receptors antibodies, or enhanced transmissibility), when rich epidemiological, clinical and/or biological data are available. In this sense, in the context of viral genomics surveillance, it is crucial to be able to rapidly detect and report such known mutations of interest in genomic sequences. As such, we developed *algn2pheno* (https://github.com/insapathogenomics/algn2pheno) [61], a tool that screens an amino acid or nucleotide alignment against a given “genotype-phenotype” database. *algn2pheno* is implemented in the routine genomic surveillance module of INSaFLU and automatically screens SARS-CoV-2 Spike amino acid alignments in each SARS-CoV-2 project against three default “genotype-phenotype” databases: the COG-UK Antigenic mutations (https://sars2.cvr.gla.ac.uk/cog-uk/) [64], the Pokay Database (https://github.com/nodrogluap/pokay/tree/master/data) [65] and a database of mutations in Spike epitope residues compiled by Carabelli and colleagues [66]. *algn2pheno* detects all the mutations in each sequence, maps the mutations to the three databases, and generates final reports with the repertoire of mutations of interest present in each sequence and their potential linkage to specific phenotypes.

This tool is also available as a standalone command line tool (https://github.com/insapathogenomics/algn2pheno) [61] and was designed for flexibility in adaptation to different pathogens and customized databases. Users must provide an amino acid/nucleotide alignment including the sequences under analysis (and the reference sequence, as mutation numbering will refer to this sequence) and a “genotype-phenotype” database in either tab-separated (.TSV) or Excel (.XLSX) format. *algn2pheno* will generate two main outputs (among other useful intermediate files): (i) a final report (tabular format with a row per sequence) that lists all the “flagged mutations” (i.e., mutations in the database that were identified in the sequences), the phenotypes associated with those mutations and a list of all the mutations in each sequence; and (ii) a binary matrix with the mutations and the “associated” phenotypes identified in all sequences. *algn2pheno* is implemented in Python 3.9 and is freely available at https://github.com/insapathogenomics/algn2pheno [61] (including usage examples and detailed output description).

### Installation and software availability

#### Easier installation using docker

Although the INSaFLU-TELEVIR website is freely available for public use, it might become limiting when robust high-speed internet is not easily available (e.g., for use in the field), when high volumes of data are uploaded (subjected to queue and computational constraints) as well as when there are any other limitations (e.g., institutional, legal, ethical, etc.), preventing the upload of sequence and/or descriptive data to external servers. It is thus essential that the INSaFLU-TELEVIR platform also be made available locally. Although fully based on open-source software, it depends on many different tools, making it relatively complex to install and configure manually. To facilitate the local installation of INSaFLU-TELEVIR, we used the docker containerization system [67] to automate the installation process, making it possible for users with limited informatics knowledge to install the system. Users just need to install docker in their system, download the INSaFLU docker from github (https://github.com/INSaFLU/docker) [68] and run a small number of commands. During this process, users can also decide not to commit to installing the virus detection module (TELEVIR) if they only need the routine genomic surveillance components (INSaFLU and Nextstrain). Although designed to be installed on a computer running a Unix-like operating system, the docker installation can also be used in a Windows platform (e.g., a laptop) using WSL, as long as sufficient computational resources are available. The minimum recommended RAM is 32G, if the virus detection module is installed, but can go down to 16G if only the genomic surveillance system is installed. One recommended use-case is the installation (e.g., by a (bio)informatician) of an INSaFLU-TELEVIR instance to be shared within an institution. Also, when a local docker instance is available, *findONTime* (see previous sections) can be used to upload reads to the local instance, and automatically create and run viral detection projects, reducing hands-on time.

#### A snakemake workflow to facilitate execution in a compute cluster

The main driver for the development of the INSaFLU-TELEVIR website was the empowerment of less capacitated laboratories, facilitating the implementation and usage of bioinformatics workflows for viral metagenomic diagnostics and routine genomic surveillance. Nonetheless, it may become cumbersome to use the INSaFLU web-based interface with a very large number of samples. Also, some laboratories may want to use the analysis pipelines available in INSaFLU through internal computational infrastructure, such as compute clusters. Moreover, the website is not a practical testbed for the development of new functionalities or integration of alternative approaches to the existing pipelines. To cater to these needs, we have implemented the functionality of the genomic surveillance module of INSaFLU as a Snakemake [69] workflow. We make use of Snakemake’s support for conda to facilitate installation of external software, and its slurm support to facilitate execution in compute clusters. The workflow is available at https://github.com/INSaFLU/insaflu_snakemake [70], including instructions on how to use it. Using the benchmark datasets described above, the INSaFLU snakemake workflow produced the same consensus sequences as the public website (Additional file 2).

## Results and discussion

Implementation of viral metagenomic diagnostics and routine genomic surveillance can be particularly challenging due to the lack of computational infrastructure, tools, and/or bioinformatics expertise. In order to face the latter challenge, we have previously developed and openly released INSaFLU (https://insaflu.insa.pt/) [19, 22], a user-friendly bioinformatics suite for virus NGS data analysis. In the present study, we developed a new module (TELEVIR) for metagenomics virus identification, and considerably expanded and reinforced its genomic surveillance modules. Currently, INSaFLU-TELEVIR (https://insaflu.insa.pt/) [22] is an open web-based (but also locally installable; https://github.com/INSaFLU/docker) [68] bioinformatics platform for virus metagenomic detection and routine genomic surveillance that can be freely accessed upon account creation (user-restricted accounts). It can handle NGS data (single and/or paired-end data) obtained from different technologies (Illumina, Ion Torrent, and ONT), and derived from diverse wet-lab protocols (amplicon-based workflows, shotgun metagenomics, etc.) and library preparation/sequencing kits. It integrates two main analytical components: i) a virus detection pipeline: from NGS reads to quality control and metagenomics virus identification and reporting; ii) a reference-based genomic surveillance pipeline: from NGS reads to quality control, mutations detection, consensus generation, virus classification, alignments, “genotype-phenotype” screening, phylogenetics and integrative phylogeographical and temporal analysis, etc. (Fig. 1). An up-to-date documentation providing extensive usage example of data upload, analysis and management, and pipeline details (complementing the code available at https://github.com/INSaFLU) [71] and an extensive tutorial on how to upload data, run analysis, and visualize/download graphical and sequence/phylogenetic outputs is available at https://insaflu.readthedocs.io/en/latest/ [72], since its first release [19].

### Usage

Following the original web interface architecture [19], the upgraded INSaFLU-TELEVIR dashboard and functionalities are organized in four main interactive tabs: Settings, References, Samples, and Projects.

The Settings menu (a new feature since Borges et al., 2018) [19] is organized, when applicable, by module (Quality Control, INSaFLU, and TELEVIR), NGS technology, pipeline step, software, and parameters. This menu should be consulted to change specific software or controlling workflows, in order to fit the desired bioinformatics pipeline to the user’s needs, sample characteristics, and/or the upstream experimental conditions. For example, the default reads end’s trimming size may be too permissive or restrictive depending on the laboratory protocol (e.g., tiling amplicon multiplex PCR) and/or on sequencing settings or bioinformatics that were applied upstream (e.g., if reads are or not already trimmed/clipped before upload). The workflow and parameters selected in the global Settings menu are applied to the user account as a whole (i.e., to new samples and projects), but specific settings can be modified later on for individual samples or projects, in the respective menus. The References menu includes publicly available sequences (from NCBI or made available in INSaFLU under permission from the authors) to be used for reference-based genome assembly through “INSaFLU” projects (see below). It has been continuously enriched with sequences relevant for surveillance of viruses of interest, namely influenza, SARS-CoV-2, MPXV, and RSV. Similarly to the first platform version, additional reference files (FASTA and GenBank) can be uploaded to the user-restricted account. The Samples menu is the main sample repository, in which NGS reads (fastq.gz format), as well as the sample contextual data (i.e., metadata table in “csv” or “tsv” format, according to downloadable templates), are uploaded (through single upload or batch upload) or deleted. This menu also provides read-quality reports, technology identification, and rapid classification data (all automatically provided after upload), as previously described [19]. The main Projects menu allows access to three types of scalable projects: TELEVIR projects (for virus detection), INSaFLU projects, and Nextstrain datasets (both for virus routine genomic surveillance). The usage and functional and reporting features of these three main analytical modules are described below.

### Metagenomics virus detection

#### TELEVIR projects—from reads to virus detection

**Fig. 3:**
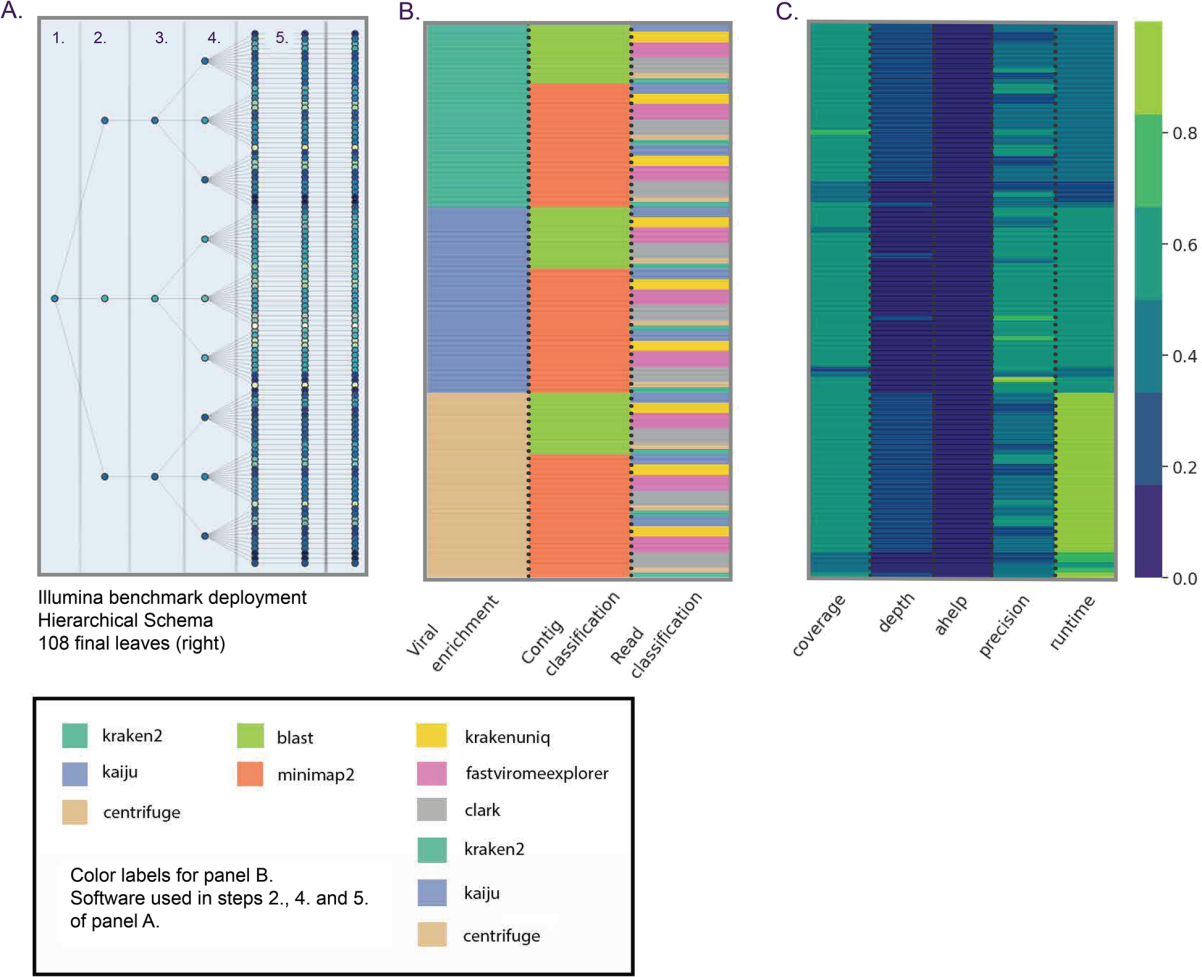
Simplified illustration of the benchmark of the virus identification pipeline (TELEVIR) module components, which is described in detail in Additional file 1. **A** Tree representation of module combinations. From left to right, sections represent pipeline steps (exemplified for Illumina) as followed at runtime: (1) Quality Control, (2) Viral Enrichment, (3) Assembly, (4) Contig Classification, (5) Read Classification. Nodes represent software, parameters, or databases compared. Color gradient corresponds to the product of four assessment statistics: mapped reads proportion, horizontal coverage, true positive rate, and completeness (proportion of hits with both read and contig evidence). Statistics were standardized by their respective maxima. **B** Heatmap representation of software benchmarked for Illumina samples, parameters not discriminated, color code at the bottom. **C** Table of individual statistics for each node, standardized across samples as in **A**. For panels **A** and **C**, darker colors = lower values, lighter colors = higher values

Our benchmarking results consolidated the expectation that there is no “one-size-fits-all” bioinformatics approach that can detect all viruses, but instead a set of “well-performing” workflows that together can potentiate the detection of clinical relevant viruses, as described in the implementation section (and detailed Additional file 1 and Fig. 3). As such, the TELEVIR dashboard was designed to accommodate this flexibility by allowing users to simultaneously select complex workflows (covering several combinations of classification algorithms, databases, and parameters) in a user-friendly manner through the TELEVIR Settings pages. Controlling workflows is done by selecting/deselecting which software (and their parameters and/or databases, when permitted) are to run at each step of the pipeline (summarized in Fig. 2). Some key pipeline steps (e.g., confirmatory re-mapping) cannot be turned OFF. Other cases are context-dependent: de novo assembly cannot be turned OFF if Contig Classification is turned ON; at least one classification step must be turned ON (Contig Classification may not be turned OFF if Read Classification is already OFF, and vice-versa).

**Fig. 4:**
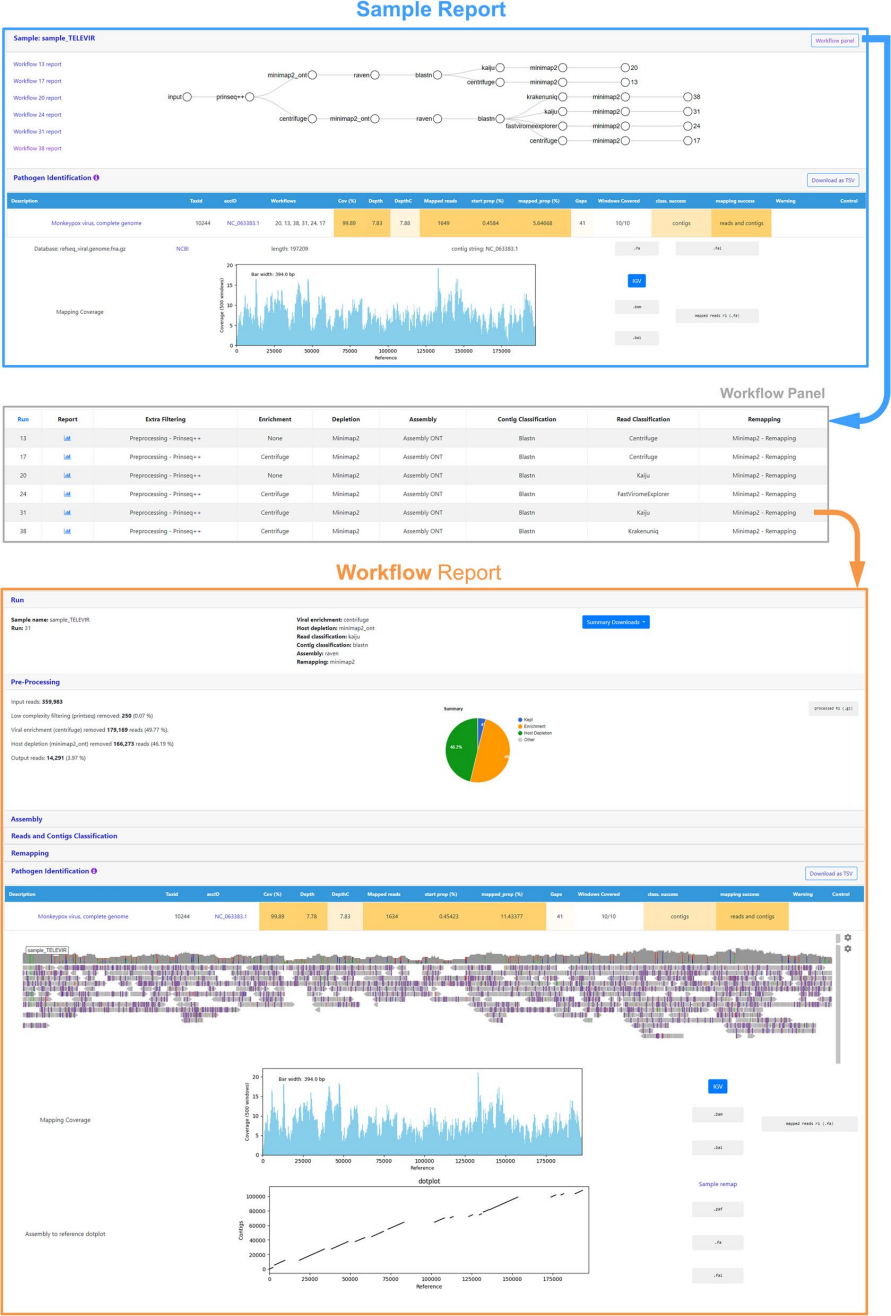
Snapshot of dashboard reports of the INSaFLU-TELEVIR bioinformatics module for metagenomics virus detection. Interactive examples are available at the https://insaflu.insa.pt/ [22] through an open “demo” account

In parallel, efforts were employed to develop and implement user-friendly (visual) solutions for output reporting. As the interpretation of metagenomics virus detection data is not a trivial task (even for users with expertise in virology and/or bioinformatics), the design of the TELEVIR output dashboard gave emphasis not only to increasing report accessibility and interpretation, but also to facilitating output navigation and promoting decision-making on the part of the users (especially relevant in clinical virology). Targeting these goals, TELEVIR reports are generated per workflow, per sample (combining several workflows), and per project (combining several samples), with a decreasing level of detail. *Workflow* reports are organized as dynamic and interactive “expand-and-collapse” panels that allow the visualization/download of relevant intermediate tabular (e.g., list of the software parameters, list of viral hits classified from reads and/or contigs) and sequence output data (e.g., reads surviving the viral enrichment and/or host depletion steps) generated throughout each workflow step (listed in Table S8; Fig. 4). Ultimately, each workflow culminates in a main report (interactive table) with a list of the detected top-viral hits, each one accompanied by several robust and diagnostic-oriented metrics, statistics, and visualizations (also detailed in https://insaflu.readthedocs.io/en/latest/) [72], provided as (interactive) tables, graphs (e.g., coverage plots, Integrative Genomics Viewer visualization, assembly to reference dotplot) and multiple downloadable output files (e.g., mapped reads/contigs identified per virus; reference sequences, BAM files, etc.) (Table S8; Fig. 4). In brief, the reported hits are identified (as detailed in the “Implementation” section), up to a user-defined maximum number of hits, as follows: reads and contigs (if available) are classified independently, then viral hits (TAXID) detected in both intermediate classification reports (reads and contigs) and/or within the top list from each side are selected for reference-based mapping against viral genome sequences present in the available databases. In summary, the main tabular report only includes viral hits (listed by the reference NCBI ACCID, with a direct interactive link to the NCBI webpage) that were classified at reads and/or contig level (“classification success”) and that had mapped reads or contigs (“mapping success”). Other viruses (TAXID) that were not automatically selected for confirmatory remapping are flagged as “Unmapped” and can be user-selected for mapping at any time through the bottom panel “Raw Classification and Mapping Summary” (which also lists hits yielding zero mapping). This functionality allows users to confirm/exclude the presence of a suspected virus (e.g., virus compatible with the animal/human clinical status) that did not meet the criteria for confirmatory remapping (e.g., due to their insufficient number of hits in the intermediate reports). *Sample* reports (interactive and downloadable tables) compile all viral hits identified in the main reports of the several workflows that were run for each sample, in which redundant hits are excluded (Fig. 4). Finally, *Project* results are provided as simple tables combining all top viral hits identified in the main reports of the several workflows that were run for all samples included in the project. Both Sample and Project reports provide direct links to the detailed reports generated at the workflow level for an enhanced sample comparison and output interpretation.

Besides the availability of multiple reports and downloadable files (summarized in Table S8), other important features were incorporated in the TELEVIR dashboard and documentation to facilitate the detection, evaluation and/or resolution of specific situations or confounding factors commonly faced during metagenomic NGS in clinical virology, in light of recent recommendations [18, 26, 27, 73].

##### Negative and positive controls

The inclusion of negative controls (e.g., pathogen-negative samples, library preparation buffers) is highly recommended to identify sources of potential contamination and detect false positive hits [26, 27, 73–77]. Indeed, viral taxa/sequences detected in the test samples that are also present in the negative run controls should be interpreted as contamination or background noise (e.g., nucleic acids present in reagents might yield false positive viral hits across test and control samples). In addition, the inclusion of positive controls (e.g., samples spiked with viruses that cannot be found in the organism or in the environment that is being investigated) is also commonly performed to control the success of nucleic acids extraction, preparation, and sequencing [18, 26, 27, 73]. As such, TELEVIR users are encouraged to create different projects for different metagenomics sequencing runs, as they are allowed to select “control” sample(s) at any time (before and after data analysis) for each project. Viral TAXIDs detected in the main report of the user-selected “control” sample(s) are automatically flagged as “Taxid found in control” in the reports of samples in the same project. This functionality is designed to facilitate the background subtraction of viral hits also found in controls.

##### False-positive viral hits

In the context of diagnostics, false-positive bioinformatics classification results can have significant consequences for patient/animal care [18, 26, 26, 73]. As such, TELEVIR reports provide specific warnings for two bioinformatics “artifacts” commonly yielding false-positive virus assignments: (i) “Vestigial Mapping” warning: when only a vestigial number of reads is mapped; (ii) “Likely False Positive” warning: when most read map in a very small region of the reference sequence, i.e., hits with high “DepthC” (mean depth of coverage exclusively in the covered regions) but low “Depth” (mean depth of coverage throughout the whole genome) and low “Cov (%)” (horizontal coverage) (specific flag criteria are detailed in Table S8). Of note, during benchmarking and testing, we noticed that both situations are often due to low-complexity regions (e.g., homopolymeric tracts or repeat regions). In this regard, an extra optional step of reads filtering by sequence complexity (using PrinSeq + +) [29] was added to the pre-processing step.

##### Multiple hits for several closely-related viruses

Cross-mapping of reads across several viruses (TAXID) with considerable nucleotide homology, such as viruses belonging to the same family, is very common in viral metagenomics. The interpretation of these cases is expected to be facilitated by the fact that the virus actually present in the sample is likely more closely related to the reference virus (TAXID) yielding the best TELEVIR mapping metrics (see Fig. S6), but extra manual inspection (namely, BLAST of mapped reads/contigs and IGV inspection) is recommended (see documentation and literature [26, 27, 73]. To further facilitate the report interpretation, viral hits included in the main reports (at both “workflow” and “sample” levels) are grouped and sorted by the degree of overlap of cross-mapped reads, as detailed in implementation. In addition, an optional and flexible step of “mapping stringency” is available to facilitate the detection of reads with high homology to the reference. Of note, by design, a true positive viral detection in TELEVIR will normally yield multiple hits for the same virus (TAXID). Two main situations justify this output: (i) the presence of segmented viruses in the sample (usually each reference segment has different ACCIDs, so they are reported as independent hits); (ii) the availability of several reference genomes (strains or variants) of the same virus in the databases. As above, in the latter situation, the virus present in the sample is likely more closely related to the reference genome (ACCID) yielding the best mapping metrics. The sorting strategy described above is expected to largely facilitate the report interpretation in these cases.

Although the INSaFLU-TELEVIR platform takes advantage of several viral reference databases, these do not cover all viruses. For instance, newly discovered or uncommon viruses or viral strains (e.g., viruses without available complete genomes in the databases) might be missing, leading to false negative results. Moreover, the ultimate goal of the TELEVIR module is to detect viruses (especially clinically relevant viruses), and not necessarily to identify the virus “strain/variant/serotype”. Once a given virus is detected, users are encouraged to perform fine-tuned analyses (e.g., consensus sequences reconstruction, mutation detection, etc.) using the classical INSaFLU projects (see below) to better characterize the virus found. Ultimately, in order to facilitate and strengthen the TELEVIR output interpretation and decision-making from the part of users, we highlight the availability of extended user guidance on how to interpret TELEVIR reports and exclude/confirm viral hits, by exemplifying “expected” metrics profiles (or combination of profiles) when there are different levels of evidence for the presence of a given virus in metagenomic NGS data analyzed through TELEVIR (https://insaflu.readthedocs.io/en/latest/) [72].

**Fig. 5:**
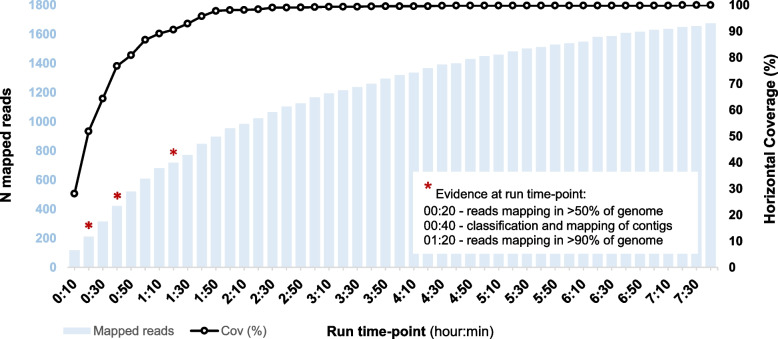
Rapid, robust, and cost-efficient diagnostics using findONTime in combination with MinION sequencing. A simulated scenario of hypothesis-free ONT sequencing using data from a MPXV-positive sample, prepared without prior viral enrichment/host depletion. The plot shows the number of reads mapping to a MPXV reference genome and the percentage of horizontal coverage at increasing time points, during the sequencing run. Reference genome identified with over 50% coverage after 20 min. Contigs mapped at the 40-min mark. Strong evidence (mapped contigs; > 90% reference genome covered by at least one read) is achieved in under 2 h (1 h 20 min)

As described in the Implementation section, apart from the development and release of the TELEVIR module, we released findONTime (https://github.com/INSaFLU/findONTime) [28], which is a complementary tool designed to run concurrently to MinION sequencing towards a more timely and cost-effective real-time metagenomics virus detection using the INSaFLU-TELEVIR platform. Indeed, by automating the input preparation (ONT reads and metadata) and TELEVIR deployment, findONTime potentiates the detection of a virus in a sample as early as possible during the sequencing run, reducing the time gap between obtaining the sample and the diagnosis, and also reducing sequencing costs (as ONT runs can be stopped at any time and the flow cells can be cleaned and reused). As a proof-of-concept exercise, we ran the findONTime over ONT data of a MPXV-positive sample (regarding the first 2022 outbreak genome described in Isidro et al. (2022)) [8] that was subjected to MinION shotgun metagenomics after DNA extraction without any virus enrichment / host-depletion laboratory treatment. As shown in Fig. 5, simulating a context of hypothesis-free ONT sequencing, this approach would allow us to get early sequence evidence for a rapid, robust, and less costly diagnosis. Indeed, although the proportion of MPXV reads was no more than 1%, strong sequence evidence was reached in less than 2 h, namely MPXV classification in both reads and contigs just after 40 min or more than 90% of MPXV reference genome covered by at least one read at 1 h 20 min of run time. findONTime can be used as a “start-to-end” solution or for particular tasks (e.g., merging ONT output files, metadata preparation and upload to a local INSaFLU-TELEVIR instance). Usage examples are provided in https://github.com/INSaFLU/findONTime#usage [28].

### Routine genomic surveillance

The surveillance-oriented component of the platform dashboard is divided into:

1. INSaFLU Projects—from reads to reference-based generation of consensus sequences and mutation annotation/screening, followed by gene- and genome-based alignments, amino acid alignments, classification, NextClade link, etc.

The COVID-19 pandemic and other recent international public health threats (e.g., the multi-country mpox outbreak, the A/H5N1 avian influenza global spread, etc.) have contributed to accelerate the “universal” access to modern sequencing technologies, in particular to portable third-generation sequencing equipments (MinION). As such, to keep following this technological revolution in the field of genomic surveillance of viral diseases, we have put particular efforts to improve and adapt the surveillance-oriented component of the INSaFLU-TELEVIR platform so that it could handle ONT sequence data of multiple viruses (besides Illumina and Ion Torrent data, as described in the first release [19]). Similarly to the Illumina / Ion Torrent pipeline, the developed ONT pipeline incorporates software for reads quality control, reference-based mapping, primer clipping, mutation calling, and consensus generation, and performed similarly to the widely used ARTIC SARS-CoV-2 pipeline (https://github.com/artic-network/fieldbioinformatics/) [53], as detailed in the benchmarking results of the Implementation section. We privileged a very smooth integration of the new ONT pipeline into the existing dashboard [19] by keeping the same user interface and features as for the existing pipeline, in order to minimize the impact on its usability and promote data analysis flexibility (e.g., ONT and Illumina samples can be run in the same project). In brief, the updated INSaFLU projects can process samples from the different sequencing technologies, which are automatically detected upon reads upload and automatically guide the pipeline to be run, without further user interaction. All upstream INSaFLU analyses (e.g., mutation annotation, alignments, and phylogenetics) and outputs (content and format) (e.g., tabular list of mutations and its annotation) were kept similar to the existing Illumina/Ion Torrent pipeline in order to facilitate sequence comparison regardless of the technology used. This harmonization and flexibility is particularly useful, for instance, in the context of routine genomic epidemiology systems with centralized data analysis, but decentralized sequencing with distinct technologies.

In addition to the integration of the reference-based genome assembly pipeline for ONT data, the INSaFLU projects were upgraded with other important surveillance-oriented (often virus-specific) functionalities and features, including (i) integration of automatic SARS-CoV-2 Pango lineage assignment (https://cov-lineages.org/pangolin) [78] using Pangolin (https://github.com/cov-lineages/pangolin) [55, 56, 79]. To better fit this dynamic lineage nomenclature, whenever new software/database versions are released (automatically checked daily), a button “Update Pango lineage” is automatically made available, so that users can re-assign all project samples using the latest software/database versions; (ii) integration of direct links to Nextclade (https://clades.nextstrain.org/) [58] for rapid and flexible SARS-CoV-2, seasonal influenza, MPXV and RSV consensus sequences analysis (at client side on browser). This feature allows INSaFLU-derived consensus sequences to be easily subjected to quality screening, lineage/clade/genotype classification, mutation exploration and other relevant analyses available at the Nextclade framework; (iii) incorporation of the newly developed “*algn2pheno*” (see implementation) for automatic screening of SARS-CoV-2 Spike amino acid alignments against “genotype-phenotype” databases of mutations of potential biological or epidemiological interest; (iv) improvement of existing features for phylogenetic trees visualization using PhyloCanvas (https://github.com/phylocanvas) [80] to easily color tree nodes and to display colored metadata blocks next to the phylogenetic trees nodes, thus facilitating integration of relevant epidemiological and/or clinical data and pathogen genomic data; and (v) inclusion of novel “expand-and-collapse” panels for an interactive report of all detected mutations (including detailed information about genome position, nucleotide change, coverage evidence, frequency, and impact at protein level), the mean depth of coverage and horizontal coverage per locus for all samples through intuitive color-coded buttons and an “algn2pheno” report of mutations of interest.

2. Nextstrain Datasets—from consensus sequences to advanced Nextstrain phylogenetic and genomic analysis, coupled with geographic and temporal data visualization and exploration of sequence metadata.

The Nextstrain (https://nextstrain.org/) [59, 60] project has played an important role in harnessing the scientific and public health value of pathogen genome data in the prevention and control of infectious diseases (well demonstrated during the COVID-19 pandemic), but also by providing up-to-date analyses of virus evolution at a global scale as well as open-sourced analytic and visualization tools. In this context, in order to promote and facilitate the real-time tracking of virus evolution (from NGS reads to the tip of the tree), we strengthened the genomic surveillance component of the INSaFLU-TELEVIR platform by integrating Nextstrain workflows for advanced analysis, visualization, and exploration of phylogenetic and genomic data together with geographic and temporal data (or any other epidemiologically relevant metadata variable). We provide the functionality of Nextstrain workflows as a new type of project named “NextStrain Dataset”. Upon creation of a new dataset, the user selects a specific Nextstrain build, either a virus-specific build (available for the four seasonal influenza, avian influenza, SARS-CoV-2, MPXV, and RSV A/B, at the time of publication) or a “generic” build that can be used for other viruses (see Implementation). For instance, a TELEVIR partner (INIA) has successfully tested the generic build with West Nile Virus data, showing its applicability to several viral threats. After creation, users can then select samples to be included in the dataset from three sources. The most common origin of the samples is reference-based assembly projects (classical INSaFLU projects), from which generated consensus sequences and associated sample metadata are automatically sent to the dataset. Users can also import sequences from the References repository (especially useful when using the “generic” build) as well as externally-provided sequences (directly uploaded as single or multi-fasta files). In the latter cases, since there is no associated metadata, default values are assumed for build-specific mandatory metadata parameters (e.g., the collection date is defined as the current date). Still, at any time, users can download the automatically generated Nextstrain metadata table, and update the default values by uploading a modified metadata file (as a tabular tsv file). To take advantage of temporal and geographical features of Nextstrain and increase their robustness, users must provide (1) “date” for all samples added to Nextstrain datasets—if no collection date is provided, INSaFLU will automatically insert the date of the analysis as the “collection date”, which might (considerably) bias (or even break) the time-scale trees; (2) “latitude” and “longitude” and/or “region”, “country”, “division” and/or “location” columns in the metadata—these values are screened against a database of geographical coordinates to geographically place the sequences in the Nextstrain map. When all samples are imported, and metadata is up to date, the user can then (re-)run the analysis and download the input consensus sequences (as a fasta file) and metadata table, as well as outputs from the build process, such as nucleotide alignments (as a fasta file), the divergence tree (as a newick file) and json file(s) that can be client-side visualized using auspice (https://auspice.us/) [81]. Consensus sequences imported into Nextstrain datasets can also be directly sent to Nextclade.

### Impact

Since its first release [19], the INSaFLU (https://insaflu.insa.pt/) [22] bioinformatics framework, which has been considerably upgraded as described in the present study, has played a pivotal role in pathogen genomics surveillance in Portugal, namely for SARS-CoV-2 (https://insaflu.insa.pt/covid19/; more than 48,000 sequences analyzed, as of October 2023) [82], for influenza (around 1000 samples analyzed in 2020–2023), and, more recently, for MPXV (around 600 samples in 2022–2023). This impact is well reflected in several works, namely in the rapid identification and characterization of emerging viral threats [8], in national [9, 83, 84] and local [85, 86] outbreak tracking, and in research studies in viral evolution [87, 88], which ultimately contributed to strengthening the integration of the genomics pathogen surveillance in public health decision-making towards infectious diseases’ prevention and control. Moreover, coupled with a portable metagenomic virus detection wet-lab protocol [21], the newly developed TELEVIR bioinformatics component could be successfully tested in proof-of-concept studies conducted by TELEVIR consortium members, under very different conditions (https://onehealthejp.eu/projects/emerging-threats/jrp-tele-vir) [20]. These exercises in a real context not only provided a good complement to the multiple tests performed during the TELEVIR pipeline development, benchmarking, and final refinement, but also introduced it as a bioinformatics resource of reference among several Public Health and Veterinary institutes across Europe. The achieved INSaFLU-TELEVIR versatility and functionality has also captured the attention of the international scientific community and key stakeholders in the field of public and animal health, leading to a considerable increase in the number of accounts created and published applications (e.g., [89–97]). This is well reflected by the multiple national and international activities that were conducted (or are being planned) to support the capacity building of several countries/laboratories in viral metagenomic detection and genomic surveillance through specific training in INSaFLU-TELEVIR. For instance, INSaFLU-TELEVIR has recently integrated ECDC training programs, through the AURORAE project (to support microbiology-related activities and capacity building focusing on COVID-19 and influenza in the EU/EEA, the Western Balkans, and Turkey) and the GenEpi-BioTrain programme in Genomic Epidemiology and Public Health Bioinformatics (with focus on strengthening knowledge and skills for use and development of bioinformatics tools in the public health context) [98]. Through the MediLabSecure project (https://www.medilabsecure.com/) consortium [99], INSaFLU-TELEVIR workshops were also recently organized to improve the surveillance and monitoring of emerging zoonotic diseases of viral origin in the Mediterranean, Black Sea and Sahel regions. The strong collaboration INSA has with countries of Portuguese official language is also prompting integration of INSaFLU-TELEVIR in emerging genomic surveillance systems in several African countries (namely, Guinea-Bissau, Angola, and Cape Verde) with training sessions, support to local installation, and other capacity-building activities having recently occurred or being planned for the next years.

## Conclusions

The early detection, characterization, and surveillance of viruses is an urgent need at a global level in the light of the continuous emergence of viral threats, as recently observed in SARS-CoV-2 and MPXV epidemics. In this context, on behalf of the EU-funded TELEVIR project, in addition to the development of wet-lab protocols for virus metagenomic detection using ONT sequencing (addressed in Fomsgaard et al., 2023)[21], we built on an existing INSaFLU platform [19] to deploy a freely accessible and user-oriented “start-to-end” bioinformatics framework for viral metagenomic detection and routine genomic surveillance: the upgraded INSaFLU-TELEVIR platform (https://insaflu.insa.pt) [22]. The present report describes: i) the development, benchmarking, and implementation of a novel virus detection module (TELEVIR), from reads and quality control to the identification of both RNA and DNA viruses; ii) the improvement and adaptation of the “surveillance-oriented” platform components to handle “multi-technology” sequence data (ONT, Illumina, and Ion Torrent) of “any” virus, from reads to quality control, mutations detection, consensus generation, alignments, genotyping (through Nextclade and pangolin), “genotype-phenotype” screening (through incorporation and standalone release of “algn2pheno” for mutation screening), phylogenetics, integrative phylogeographical and temporal analysis (through incorporation of Nextstrain). In addition, we further developed an innovative complementary standalone tool (“findONTime”) for real-time automated INSaFLU-TELEVIR data upload and analysis during MinION sequencing, to promote a more timely and cost-effective sequencing by reducing, as much as possible, the time gap between obtaining the sample and viral detection and characterization. Although it was challenging to develop this work in the context of a pandemic and a research field in continuous technological evolution (microbial genomics and metagenomics), the final platform functionalities benefited from the real-time software and data sharing at the international level [100–102], leading to the integration of cutting-edge and state-of-the-art bioinformatics features and resources [57, 79, 103]. In addition, we should highlight that the TELEVIR workflows for data analysis and reporting (as well as the tutorial and guidance provided in the extensive online Documentation) were strongly inspired and tried to cover state-of-art recommendations for the introduction of metagenomic next-generation sequencing in clinical virology [18, 26, 27, 73]. As such, the module does not propose to introduce yet another algorithmic approach to the already brimming field of metagenomics classification. Rather, TELEVIR focuses on harnessing the diversity of existing approaches in the interest of the end-user by allowing the simultaneous deployment of several workflows. Indeed, the infrastructure proposed makes available to the non-technical user the aforementioned methodologies, along with several databases, and this versatility is couched within a framework that promotes cross-validation between different methodologies. Finally, the presentation of final reports is provided in a way that is un-categorical, treating metagenomics hits as investigative leads rather than sure-fire results. We believe this latter point is particularly important, since it prevents an over reliance on automated diagnosis, emphasizing the importance of domain knowledge and informed interpretation. Regarding this topic, it is crucial to acknowledge that the interpretation of clinical relevance of the metagenomics hits, which goes beyond their bioinformatics confirmation, remains another challenging topic that the scientific community still needs to address towards a more straightforward and routine application of metagenomic in clinical settings. Ultimately, although the INSaFLU-TELEVIR platform offers multiple bioinformatics capabilities and opportunities, it also bears noteworthy limitations. The local version is more flexible than the online tool in terms of maximum input size, but it might pose installation challenges and the integration of new analytical modules might not be straightforward to external users, as it does not rely on popular workflow managers, such as Nextflow. Additionally, although our simulation yielded positive outcomes, the detection and genome reconstruction of unknown viruses or very divergent variants might be challenging, due to the inherent public database diversity constraints and workflow architecture, which is more tailored to pathogen’s diagnosis/surveillance rather than discovery. Efforts directed towards addressing the above-mentioned challenges are ongoing, such as the integration of protein-based taxonomic tools to potentiate divergent virus discovery, the incorporation of targeted reference screening to facilitate the direct search for viruses that may be clinically suspected, the flexibilization of mapping configuration and reporting to further mitigate false-positive hits attributed to cross-mapping. In summary, the achieved accessibility, the “pan-viral” nature, and the functionality of INSaFLU-TELEVIR have captured the attention of the scientific community, leading to a considerable increase in the number of users, and to the engagement of the INSaFLU-TELEVIR team in multiple national and international activities (conferences, training programs, networks, and projects) to strengthen the capacity for genomic epidemiology and “One Health” bioinformatics of laboratories/countries conducting surveillance of virus with impact on human and animal health. INSaFLU-TELEVIR is a free web-based, and locally installable, platform available at https://insaflu.insa.pt/ [22]. 

### Supplementary Information

**Additional file 1.** Benchmark of the INSaFLU-TELEVIR pipeline for virus detection (TELEVIR): Resources, Workflow details, Benchmark and Implementation.
**Additional file 2.** Benchmarking of INSaFLU against commonly used command line bioinformatics workflows for SARS-CoV-2 reference-based consensus generation (amplicon-based Illumina and ONT data), and validation of the INSaFLU snakemake pipeline.
**Additional file 3: Supplementary figures 1-8.**
**Additional file 4: Supplementary tables 1-8.**

## Data Availability

The INSaFLU-TELEVIR platform is available as a free online tool (https://insaflu.insa.pt) [22] and as a locally installable version (https://github.com/INSaFLU/docker) [68]. A snakemake pipeline to run the surveillance-oriented reference-based genome assembly INSaFLU component (for both ONT and Illumina) is available at https://github.com/INSaFLU/insaflu_snakemake [70]. All INSaFLU documentation (latest) for each module is provided at http://insaflu.readthedocs.io/ [72]. Users can also walkthrough a INSaFLU-TELEVIR “demo” account available at the login page. The software source code is available under the GNU license—GPL 2.0 (https://opensource.org/licenses/GPL-2.0), from the GitHub repository at https://github.com/INSaFLU/INSaFLU [71]. Raw read data used for INSaFLU (Additional file 2) and TELEVIR (Additional file 1) modules benchmark was deposited in the European Nucleotide Archive (ENA) (BioProjects PRJEB67829 and PRJNA1081297, as detailed in Table S4). The code used to perform the exploratory data analysis (EDA) of the TELEVIR metagenomics classification benchmark is available at 10.5281/zenodo.8428029 [104]. Artificial sequence datasets generated to assess mapping performance are available at https://zenodo.org/doi/10.5281/zenodo.10731592 [105].
