# Supplementary material for: INSaFLU-TELEVIR: an open web-based bioinformatics suite for viral metagenomic detection and routine genomic surveillance
Source: Genome Med. 2024 Apr 25;16:61. doi: 10.1186/s13073-024-01334-3 (PMC11044337; doi:10.1186/s13073-024-01334-3)
Supplement: Supplementary file 1 — Additional file 1. Benchmark of the INSaFLU-TELEVIR pipeline for virus detection (TELEVIR): Resources, Workflow details, Benchmark and Implementation. Additional file 2. Benchmarking of INSaFLU against commonly used command line bioinformatics workflows for SARS-CoV-2 reference-based consensus generation (amplicon-based Illumina and ONT data), and validation of the INSaFLU snakemake pipeline. Additional file 3: Supplementary figures 1-8. Additional file 4: Supplementary tables 1-8. [file 13073_2024_1334_MOESM1_ESM.zip › Additional file 1.docx]

**Additional file 1**

Here we provide a detailed summary of the resources and implementation details of the benchmark of the INSaFLU-TELEVIR pipeline for virus detection (TELEVIR). This summary is split into four parts: Resources, Workflow details, Benchmark and Implementation. The first two sections regard the construction of the pipeline. While only a subset of resources was eventually implemented into the web platform (<https://insaflu.insa.pt/>), most implementation details are common to benchmark and the released pipeline, with the exception of some features included after (and usually due to) benchmarking. These latter additions are flagged. The last two sections, Benchmark and Implementation, describe the results of our benchmarking study, our rationale for selection of software, and the details of the implementation in the web version (<https://insaflu.insa.pt/>), at the time of publication.

1. Resources

1.1. Description of Software benchmarked

1.2. Description of Databases benchmarked

1.3. Taxonomic indexing

1.4. Description of benchmark FASTQ samples

2. Workflow details

2.1. Reads pre-processing

2.2. Viral Enrichment and Host Depletion

2.3. *de novo* Assembly

2.4. Read Classification

2.5. Contig Classification

2.6. Confirmatory Remapping and Reporting

2.6.1. Processing and harmonizing classification outputs

2.6.2. Sorting and merging classification outputs (Intermediate Classification report)

2.6.3. Reference sequence selection (Final Report)

2.6.4. Grouping and Sorting mapping results in the Final reports (optional feature)

3. Benchmark

3.1. Layout

3.2. True positive criteria

3.3. Metrics and Statistics

3.3.1 Final Report - Sorting and reference sequence selection

3.3.2 Workflow comparison

3.4. Results

3.4.1. Final Report - Sorting and reference sequence selection

3.4.2. Workflow comparison - Preliminary analysis

3.4.3. Workflow comparison - assessment of software performance

3.4.4. Workflow comparison - overall assessment

3.4.5. Confirmatory Mapping *versus* Nucleotide divergence - simulation exercise

3.4.6. Reporting Statistics

4. Implementation

4.1 Software and Database selection

4.2 Final Report

**TELEVIR benchmark and implementation**

**1. Resources**

**1.1. Description of Software benchmarked**

The software used during benchmark and implementation is listed in Supplementary Table 1 and described below.

*Centrifuge*

Classification with Centrifuge (version 1.0.4) [1] is based on exact matches of short sequences against a compressed reference index. The compressed index is built from the *NCBI Refseq Complete Genomes* database as part of normal installation.

#### *Clark*

Clark (version 1.2.6.1) [2] is a taxonomic classifier that relies on short nucleotide k-mer classification. It uses a compressed index database containing unique k-spectra specific to target sequences. For the current comparison, the default execution mode was used.

#### *Kraken2*

Kraken2 (version 2.0.8-beta) [3] is a classifier designed to improve the large memory requirements of the former version of Kraken [4], resulting in a reduction of in general 85% of the size of the index database. Kraken2 uses a probabilistic, compact hash table to map minimizers to the lowest common ancestors (LCA), and stores only minimizers from the reference sequence library in its reference index.

#### *Kaiju*

Kaiju (version 1.7.3) [5] is a taxonomic classifier that assigns sequence reads using amino acid-level classification. Sequence reads are translated into six possible open reading frames (ORFs) and split into fragments according to the detected stop codons. Classification with Kaiju can be performed using two settings, both based on an adjusted backward alignment search algorithm of BWT [6]. In the default greedy mode Kaiju assesses six possible ORF’s using the amino acid scores of Blosum62 [7] to obtain the highest scoring match.

*Diamond*

Diamond (v.2.0.7) [8] is a taxonomic classifier that relies on the double indexing of reference and query databases to accelerate sequence matching. The latest version of Diamond optimizes its baseline algorithm by taking advantage of modern computer architecture and parallel processing, and introduces several options to control the sensitivity / runtime balance. It is a DNA-to-protein aligner.

*FastViromeExplorer*

FastViromeExplorer (v1.3) [9] uses Kallisto [10], a pseudoalignment based approach originally developed for alignment and quantification of RNA-seq data, to rapidly map short metagenomic reads to a reference virus database. FastViromeExplorer filters the alignment results based on minimal coverage criteria.

*deSAMBA*

DeSAMBA [11] is a long-read classification approach based on a pseudo alignment algorithm (approximate match block, SAMB). Reads are initially partitioned into “seed blocks”, segments that reduce the probability of false positive matches. A Unitig-BWT index is then used to find maximal exact matches between seed-block suffixes and reference sequences.

*KrakenUniq*

KrakenUniq (v0.7.3) [12] is a classification method that builds on Kraken2’s efficient probabilistic and compact hash table, complementing it with a method to ascertain false positive hits based on a unique k-mer count per identified taxon. Unique K-mer counts are estimated using the algorithm HyperLogLog.

*Blastn*

The well known Basic local alignment tool (v.2.21.0) [13].

*Minimap2*

Minimap2 (v.2.24) [14] is a general purpose pairwise-alignment tool that follows a seed-chain-align algorithm to find the best and longest local alignments from initial exact matches.

**1.2. Description of Databases benchmarked**

The sequence databases used for Classification during benchmark of TELEVIR module used are listed in Supplementary Table 2 and described below.

*Virosaurus*

Virosaurus is a database of Eukaryotic viruses designed to offer a non-biased, automatized and annotated source for clinical metagenomics studies and diagnosis. Virus sequences belong to genera able to infect eukaryotes as per ViralZone (<https://viralzone.expasy.org/655>) and are filtered for complete genomes. Downloaded on the 9th of January 2023.

*Kraken2 Metagenomic Virus Database*

A database built from the integration of a classification tree built from the over 700k metagenomic viruses from the JGI IMG/VR database [15] with the NCBI taxonomy dataset. Downloaded on the 9th of January 2023.

*NCBI Refseq Complete Genomes*

Viral genomes were downloaded from the NCBI RefSeq database by iterating through the contents of the ‘[ftp.ncbi.nih.gov://genomes/refseq/viral/](http://ftp.ncbi.nih.gov/genomes/refseq/viral/)’ for the latest release. Downloaded on the 9th of January 2023.

*NCBI taxonomy*

The standard nomenclature and classification repository for the International Nucleotide Sequence Database Collaboration (INSDC), comprising the GenBank, ENA (EMBL) and DDBJ databases. Downloaded on the 9th of January 2023.

Swissprot

The viral subset of the Swiss-Prot UniRef90 protein database (Suzek et al. 2007) was downloaded from NCBI blast database (<https://ftp.ncbi.nlm.nih.gov/blast/db/FASTA/swissprot.gz>) on the 9th of January 2023.

Of note, some classifier tools, such as Kraken2 and Centrifuge, are distributed with pre-compiled reference databases. These are generally the NCBI Refseq complete genomes dataset, with some modifications (i.e. dustmasking in case of Centrifuge) and additions.

**1.3. Taxonomic indexing**

*Accession to TAXID map*

Nucleotide sequences from Refseq, Virosaurus, Kraken2 and Centrifuge Refseq databases were parsed for sequence identifiers (ACCIDs). The respective taxonomic identifiers (TAXIDs) were queried using the NCBI Entrez Direct utilities (<https://www.ncbi.nlm.nih.gov/books/NBK179288/>), by extracting AccessionVersion and TaxId entries of ACCID records in the Entrez Direct Nucleotide database (nuccore, <https://www.ncbi.nlm.nih.gov/books/NBK3837/#:~:text=The%20Entrez%20system%20comprises%2039%20molecular%20and%20literature%20databases>).

*Protein accession to TAXID map*

We downloaded the NCBI protein to taxonomic description database files 1 through 12 (prot.accession2taxid.FULL.{x}.gz) from <https://ftp.ncbi.nlm.nih.gov/pub/taxonomy/accession2taxid/>. Following the download of protein sequence databases (see Databases section), we parsed files for protein accession IDs and merged them to the NCBI database in order to generate a database-specific accession-to-taxid map file for Swissprot.

**1.4. Description of benchmark FASTQ samples**

As input, we used raw, untrimmed read datasets (retrieved from literature [16] or provided by TELEVIR partners <https://onehealthejp.eu/jrp-tele-vir/>) well characterized by RT-PCR, and reflective of the prospective use-cases in the range of viral and host diversity, and the range of viral abundance, as measured in PCR cycle threshold (Ct) or number of reads, depending on the information available (Supplementary Table 4).

*ONT data*

Our ONT read dataset consisted in: five SARS-CoV-2 positive human samples (host-depleted against the hg38 human reference); three influenza A/H1N1 positive human samples; one cattle sample positive for Epizootic hemorrhagic disease virus (EHDV); three Bluetongue virus samples (BTV) samples, two collected from sheep, one from a Culicoides specimen; one caprine Herpesvirus 1 samples, one cervid Herpesvirus 2 sample and one bovine herpesvirus 5; one Bovine viral diarrhea and one Parainfluenza 3 Bovine samples; three Human Monkeypox samples (un-depleted).

*Illumina data*

Our Illumina data set consisted of two sets of samples. The first set of samples was retrieved from [16], as it is a standard data set that has been well described and used previously in benchmarking tests [16,17]. Generation of the metagenomics data sets has been described in deVries et al. (2021). Samples derive from human patients with encephalitis or respiratory complaints and include various tissue types (see deVries et al. 2021 for details [16]) and viruses. Of note, these samples were spiked with equine arteritis virus (EAV) and phocine herpesvirus 1 (PhHV-1), as internal positive controls, and human reads were excluded after mapping against human genome GRCh38. The second set was provided by TELEVIR partners and reflective of prospective use cases. This consisted in five A/H1N1 positive human samples; three samples positive for Epizootic hemorrhagic disease virus (EHDV), one from cattle and two from a Hamster cell line; three Bluetongue Virus (BTV) samples, two collected from sheep, one from a Culicoides specimen.

**2. Workflow details**

The TELEVIR module consists of a pipeline with the following analytic components: Quality Control, Viral Enrichment, Host Depletion, Read Classification, Assembly, Contig Classification, and Confirmatory Remapping and Reporting, as described in the Implementation section and Figure 2 of the main text. More details about each step are presented below.

**2.1. Reads pre-processing**

This procedure is the same as the default pre-processing performed on sample upload to the INSaFLU-TELEVIR platform. For Illumina/Ion Torrent data, Trimmomatic (v. 0.36) [18] is applied with the following settings: SLIDINGWINDOW:5:20 LEADING:3 TRAILING:3 MINLEN:35 TOPHRED33. For ONT data, Nanofilt (v.2.6.0) is run with the following settings: -q 8 -l 50 --headcrop 30 --tailcrop 30 --maxlength 50000). Additionally, in the TELEVIR pipeline, reads can be filtered for complexity at user demand using the DUST and Entropy approaches as implemented in the software PrinSeq++ [19]. Of note, this additional step was implemented following observations derived from the results of the benchmark presented below and did not contribute to the benchmarking results presented in this article.

**2.2. Viral Enrichment and Host Depletion**

These steps are performed using classification and read mapping software, for enrichment and depletion respectively. Following mapping, read names of classified or successfully mapped reads are extracted from the respective reports. In the case of Viral Enrichment, fastq files are filtered *for* these identifiers, using the software seqtk v1.3 (<https://github.com/lh3/seqtk>, command: seqtk subseq); for Host Depletion, fastq files are filtered for read identifiers marked as unclassified, or unmapped (not in the reports), using the same procedure.

**2.3. *de novo* Assembly**

Assembly is performed using the subset of reads that survive the Pre-processing, Viral Enrichment and/or Host Depletion steps. Assembled contigs are filtered for a minimum length of 50 bp and a maximum length of 50000 bp.

**2.4. Read Classification**

Read classification outputs are parsed for query and target sequence ids. Target sequence columns are labeled in a software specific manner for accession ID (ACCID), taxonomy ID (TAXID), protein ID or protein accession ID. For software Blast we also extract the length of individual mappings, used for sorting references (see below).

**2.5. Contig Classification**

At the Contig Classification step, we benchmarked Blastn and Minimap2 (and implemented the former), two read alignment software, as classifiers. For Blastn, the tabular mapping output is requested and the first and second columns (qseqid and sseqid) are extracted. For Minimap2, the first and fifth columns (ACCID and qseqid, pairwise alignment format PAF) of the output file are extracted.

**2.6. Confirmatory Remapping and Reporting**

Metagenomics classification software deployed in the course of a TELEViR metagenomics workflow are couched within a common analysis and reporting pipeline. Within this framework, Classification Steps (Read Classification and Contig Classification) are required to be interoperable, their output indistinguishable to downstream steps. In the next section we describe the process of harmonizing different software output.

**2.6.1. Processing and harmonizing classification outputs**

Metagenomics classification software attribute identifiers to successfully classified input material. However, different software may provide different attributes as output: KrakenUniq outputs protein identifiers; Diamond outputs protein accession identifiers; Centrifuge, Fastviromeexplorer and Blast output nucleotide ACCID; Clark, Kaiju, Kraken2 output TAXIDs. In order to ensure uniformity of the output among classifiers, we selected to convert all identifiers to TAXID. This decision comes at the cost of the specificity of software providing accession level detail, but the one-to-many relationship between TAXID and ACCID prevents us from inferring accession from taxonomy alone.

ACCID are converted to TAXIDusing the NCBI Entrez-Direct Utilities esearch (Taxonomy database). Protein identifiers (KrakenUniq) are converted to ACCID using the seqid2taxid.map file provided by KrakenUniq and then to TAXID as above. Protein accession identifiers (Diamond) are directly converted to TAXID using the Refseq Protein Full accession2taxid database (<https://ftp.ncbi.nlm.nih.gov/pub/taxonomy/accession2taxid/>), downloaded as part of TELEViR installation (see metadata preparation above).~

**2.6.2. Sorting and merging classification outputs (Intermediate Classification report)**

Processed classification outputs (above) consist of a set of read / contig to TAXID tuples. In the case of outputs in SAM format, the length of individual mappings may also be available. Viral hits (i,e, TAXID) may be present in up to two reports: reads only, reads and contigs or contigs only.

For the purposes of confirmatory remapping (next step), we proposed to sort TAXID prior to selection, based on mapping information. We benchmarked a simple sort based only on the number of reads mapped against an algorithm that takes into account both sources of classification (reads and contigs).

Algorithm:

- i. reports are compressed as counts per TAXID. If mapping length is available, it is summed by TAXID.
- ii. reports are sorted by number of counts (and mapping length if available) in decreasing order.
- iii. shared TAXID are extracted, retaining relative order.
- iv. while both reports remain non-empty, the top hit of each report is removed and added to the shared report alternatively until either is empty.
- iv. remaining reported references are appended last.

Results of the benchmark are presented below. The algorithm described here was used for benchmarking software and implemented in the online platform INSaFLU-TELEVIR.

**2.6.3. Reference sequence selection (Final Report)**

TELEVIR selects a default maximum number of TAXID from the head of the sorted Intermediate Classification Report (as described above) for confirmatory remapping and inclusion in the Final Report table. The default maximum number of TAXID to select for confirmatory remap was defined through benchmark (see **3.4.1**). TELEVIR then considers all nucleotide sequences indexed to these TAXID among nucleotide databases (see accession to taxonomy database generation and available databases sections). In the case of duplicate ACCID, those present in the NCBI Refseq and Virosaurus databases are given preference (in that order in case they comprise the sources). TELEVIR selects up to a user-defined reference (default: 12) sequences for confirmatory remapping per TAXID. If this number is exceeded, then the user-defined number of sequences is selected at random. This number was selected to cover the range of expected segments in segmented viruses because reference segments normally have different ACCIDs, so they are reported as independent hits. We note that ACCID may also correspond to different variants of the same species and that there is currently no convention to tell them apart.

**2.6.4. Grouping and Sorting mapping results in the Final reports (optional feature)**

In order to simplify the final reports (per sample and per workflow) and facilitate the identification of potential false positive hits (often arising from cross-mapping with true positive hits), viral references are grouped and placed together by mapping affinity, as measured by shared mapped reads, as follows: i. an *ACCID x reads* presence-absence matrix is built (multiple mapping information disregarded); ii. reads mapping against 5% or less of references in the report are filtered out; iii. a maximum of 100000 reads are kept by sampling matrix columns without reposition; iv. a dissimilarity matrix is constructed using the jaccard distance metric (scipy.spatial.distance python package), and a tree is constructed from the distance matrix (Bio.Phylo.TreeConstruction python package) using a neighbor joining algorithm.

The summary statistics “private_reads” and “pairwise_max_reads” are then extracted for each node, calculated as:

- private_reads : the proportion of reads found to map only to descendents of each node.
- pairwise_max : i. the reciprocal proportion of reads mapped between every pair of descents. ii. the maximum of each pair. iii. the minimum across pairwise maxima across pairs (i.e. all samples must share at least X% (user-defined) of their reads with another sample among descendents of the same node).

Grouping threshold statistics, i.e. the threshold minimum proportions of “private_reads” and “pairwise_max_reads”, are used to select among inner tree nodes, keeping the closest to the root when nested. Nodes are sorted by total number of private reads. In the final report, references are sorted by parent nodes, references within nodes are sorted by mapping coverage. Finally, references manually selected for remapping but that resulted in no mapped reads or contigs are appended last.

**3. Benchmark**

**3.1. Layout**

For ONT data, we ran 117 combinations (i.e., different software, reference databases and/or parameters) on 22 samples (a total of 2574 runs). For Illumina data, we ran 108 combinations on 23 samples (2484 total runs). Workflows comprise all combinations of available software by module and respective databases (Supplementary Tables 1-3). For some software we also compared a limited combination of parameters (Supplementary Tables 4 and 5).

**3.2. True positive criteria**

All samples included in this study had previously been verified to carry viral material. Validation varied between sources in method (majority through PCR, some using metagenomics approaches) and in output format. Our assessment of a correct match (i.e., detection on the expected virus) reflects a compromise given these sources and the output of the TELEVIR pipeline, which comprises ACCID, TAXID and description (Supplementary Tables 6-7), as follows:

- Our initial assessment metadata for each sample consisted in lists of varying lengths of TAXIDs, ACCIDs and taxonomic descriptions of viral hits (see Supplementary Tables 6-7).
- Following Classification, unique TAXID hits for each sample were further scrutinized by hand in order to improve true positive metadata: assessment reached at most the species level, with strain information being disregarded. For example, monkeypox and influenza classifications, were accepted as true positive whenever the words “monkeypox” or “influenza” were found in the hit description, respectively; Host information in the description was also used, if useful to identify the expected virus, when available except in the case of Malignant Catharral Fever (sample MCF-P6_replig), where any herpesvirus 1 was deemed as a true positive, as indicated by the sample provider.

Post matching to our database, classification information was compared to sample metadata according to the following rules:

- returned True if TAXID matching was exact;
- returned True if ACC ID matching was exact;
- returned True if the description provided matched the viral hit description using a reciprocal case-insensitive string membership test;
- Return False otherwise.

**3.3. Metrics and Statistics**

In order to evaluate the benchmarking results, we chose a set of metrics to best reflect robustness, performance as well as output interpretation [20]. We considered firstly the proportion of correct references identified, i.e. precision, in intermediate and final reports. Secondly, for the user point of view, we considered a subset of the reporting statistics: horizontal coverage (with and without gaps), the relative proportion of mapped reads relative to input, and whether the references were identified using a single source (reads or contigs) or both. This latter parameter permits us to evaluate not just classifiers individually, but the performance of software at the enrichment, assembly and classification steps combined, and is a useful metric in user confidence.

**3.3.1 Final Report - Sorting and reference sequence selection**

The Final Reports consist of a detailed analysis of a subset of the TAXID classifications obtained by classification methods (see Intermediate and Final report construction sections above). We studied the influence of the sorting algorithm and threshold number of TAXIDs selected for remapping in the composition of final reports in terms of precision and recall, across pipelines, calculated at the level of intermediate reports (above). In the following description, Final reports are considered as samples of the respective intermediate report (selected from head after sorting - see above).

Sample size ranged between 1 and 20 TAXIDs. Precision was calculated as the proportion of true positive TAXIDs identified in the sample divided by the number of total TAXIDs identified in the intermediate report. At this level we also estimated recall as the proportion of true positive TAXIDs divided by the number of distinct TAXIDs actually in report, and F1, the harmonic mean of precision and recall [21–23].

Results were pooled across all workflows in the benchmark, by technology.

**3.3.2 Workflow Comparison**

In order to compare software and workflow performance we considered a number of statistics with potential value for exploratory data analysis and statistical inference:

- Cov (%): horizontal coverage (i.e., percentage of the reference sequence covered)
- Depth: mean depth of coverage throughout the whole genome
- DepthC: mean depth of coverage exclusively in the covered regions
- start prop (%): number of mapped reads divided by the number of input reads (after QC)
- class. success: indication of whether the TAXID was selected for mapping after reads and/or contigs classification
- mapping success: indication of whether reads/and contigs successfully mapped against the TAXID representative references sequence

The proportion of mapped reads (relative to input), coverage and depth were standardized by dividing by the respective maximum across workflows for each sample. We then proceeded to estimate average values for each workflow across samples using a bootstrap procedure with 100 draws.

Additionally, workflow precision was calculated at the level of final reports as the proportion, at each run, as the number of true ACCIDs identified divided by the total number of ACCIDs in the final report.

**3.4. Results**

**3.4.1.** Final Report - Sorting and reference sequence selection

Overall, for both ONT and Illumina technologies, we found that sorting by the number of reads and contigs mapped provided better results than mapping using just reads (Fig. S1). Overall, recall rises slowly but reaches high levels above 15 (0.95), independently of the sorting strategy used. However, precision, i.e. the proportion of true positive TAXIDs selected, remains low (average below 50% for both technologies) when sorting by mapped reads only. When using the more complete sorting algorithm precision rises to 60% and 70% on average for ONT and Illumina data, respectively, by threshold of 14.

Based on these results, we set the default threshold number of selected TAXIDs to 15 for the remaining analyses.

**3.4.2. Workflow comparison - Preliminary analysis**

A preliminary analysis found coverage (horizontal coverage - COV %, see section 3.32), the proportion of input reads mapped and depth (both estimates) to be highly correlated for both technologies (see Fig. S2). As such we chose to use coverage in the following analyses. The proportion of true positives identified using contigs, precision and coverage were found to have low correlation. Considering that each describes different aspects of the pipeline, the following results are described in terms of these statistics: coverage, the contribution of contigs (abbreviated to “ahelp”), and precision.

Runtime was not taken into account for concerns regarding the uneven effect on this metric of concurrent resource use in the deployment environment. The RVDB database, due to its size, resulted in extended runtimes and sometimes out-of-memory errors during early testing. Despite its quality, it was deemed unsuitable for the point-of-incidence targets of TELEVIR and was excluded from the benchmark early in development.

**3.4.3. Workflow comparison - assessment of software performance**

We focused on the results as pooled across all samples included in the benchmark, by technology.

**Viral enrichment**

Three software (Kraken2, Centrifuge and Kaiju) were benchmarked for Illumina Viral Enrichment. For ONT, three software were also benchmarked: Centrifuge, KrakenUniq and Kaiju (see Supplementary Table 3).

For ONT data, Kaiju produced the best average proportion of reads mapped, but Centrifuge produced the highest maximum. However, we could not find significant differences between the three software tested when considering coverage, precision or the proportion of mapped reads, except that Centrifuge obtained the least variable results when these variables were analyzed in combination. We note that these results are conditioned on the performance of the workflow downstream. For illumina data, Kraken2 achieves significantly better precision than kaiju or centrifuge. However, it ranks last in terms of coverage and the proportion of reads mapped. The proportion of correct classifications obtained using contigs is also higher for Kraken2, but the distributions largely overlap (Fig. S3).

**Contig Classification**

Two software were benchmark for Contig Classification: Nucleotide Blast and Minimap2 (see Supplementary Table 3).

We looked at the average proportion of true positives identified using contigs per run as a measure of the performance of Assembly and Contig Classification steps combined. For ONT data, this metric was generally low, averaging 3%, but still unambiguously better results were obtained using Blastn over Minimap2 (using either Virosaurus or Refseq Complete Genomes). For Illumina data, the software used to classify contigs had no impact over precision. However, like for ONT data, the proportion of positive classifications obtained using contigs was significantly higher using Blastn (Fig. S3). The overall proportion of references identified using contigs was much higher than for ONT technologies, averaging 60 %.

**Read Classification**

Six software were benchmarked for Illumina Read Classification: Kraken2, Clark, Kaiju, KrakenUniq, Centrifuge and FastViromeExplorer. For ONT, seven software were benchmarked: Centrifuge, KrakenUniq, Diamond, deSamba, FastViromeExplorer, Clark and Kaiju (see Supplementary Table 3).

Within our benchmark TELEVIR framework, precision is calculated from the combination of two sources: read and contig classification (see above). Performance is thus impacted by these two steps and its analysis does not pertain to the performance of each software in isolation but in combination with software in other modules.

For ONT data, Centrifuge obtains the best results, followed by Krakenuniq, diamond and desamba. Clark, Fastviromeexplorer and Kaiju obtain the highest rate of false positives, and the rates are significantly different (see Fig. S3). Looking at the proportion of true positives in final reports that were identified using reads, we find that this order changes, with Kaiju obtaining better results than centrifuge, but without the significant differences observed for precision. When looking at the degree of concordance between contig and read classifiers, we find that the initial order is partially recovered.

For Illumina data, we could not find significant differences in software performance in terms of precision, impact on final report composition or congruence with contig classification. Overall, Kraken2 achieves better average precision, followed by Clark, Kaiju and Krakenuniq. Clark results show high variance relative to other software (Fig. S3).

**Complete workflow**

Finally, we analyzed the three modules for which different software were tested (Viral Enrichment, Contig Classification and Read Classification) together (Fig. S4).

For ONT data, focusing on the precision of final reports, Centrifuge obtains the better results independently of the software used in the other modules, followed by Krakenuniq. Regarding contig classification, Minimap2 displays an increase in precision relative to Blastn, but is also more variable in its results. When considering the product of precision, evidence completeness (the proportion of true positive references identified using both reads and contigs), and the proportion of reads mapped, then, for Contig Classification, Blastn does perform better, consistent with the results above. At the Read Classification step, Centrifuge and Krakenuniq provide the best results, followed by Diamond. KrakenUniq performs well independently of software / parameter combinations in other modules.

For Illumina data, focusing on precision we again find the positive impact of Kraken2 in the Viral Enrichment step, and the best results are obtained in combination with Minimap2 in the Contig Classification step and either itself or Kaiju as Read Classifiers. While Clark obtains good average results we note that the variance is again very wide relative to other software. Overall, the combination of Kraken2 / Centrifuge in either Viral Enrichment or Read Classification steps performs well. When analyzing precision, horizontal coverage and the proportion in combination with true positives identified using contigs, then Kraken2 is the clear favorite in any setting. Again Clark obtains high average values with very high variance.

**3.4.4. Workflow comparison - overall assessment**

Precision is generally low across workflows and technologies, with the best workflows achieving an average of 58 and 41 % for Illumina and ONT technologies respectively.

When constraining analyses to Human samples we find that the relative workflow performance remains the same, but that for Illumina data / workflows Final Reports across pipeline combinations display a significant reduction in precision (from 58 % to 40 %, results not shown).

False Positive hits were mostly due to cross-mapping to closely related species, frequent notably among herpesvirus, coronavirus and influenza. In some cases our analysis indicates the presence of co-contaminants, such as in the case of the Adenovirus illumina sample cs021, which may carry a herpesvirus 4, previously unreported. Endogenous retroviruses, namely Human RD113 and K113 retroviruses, are frequently retrieved among human samples.

The above results concern all samples considered in the benchmark. We found no significant difference when subsetting for human samples (results not shown).

**3.4.5. Confirmatory Mapping *versus* Nucleotide divergence - simulation exercise**

One of the most challenging topics when developing a novel bioinformatics workflow for metagenomics diagnostics is to address its capability to detect unknown viruses or very divergent variants, particularly those not represented in the commonly used curated viral reference sequence databases (like the non-redundant NCBI’s RefSeq database or the Virosaurus) [24]. Indeed, the effectiveness and likelihood of detection is dependent on multiple factors (e.g., database diversity per TAXID, amount of viral reads in the sample, their distribution across the genome, etc), each one with several layers of complexity, that cannot be reduced to a simplistic threshold of nucleotide identity. For instance, the genetic polymorphism is not evenly distributed throughout a viral genome, so a low average identity between a true hit and the reference sequences does not necessarily translate into false negatives (as reads in conserved regions are still likely to map to “divergent” references). Despite this context, we performed an exercise to assess the influence of incremental genetic divergence on the efficacy of the Read classification and Mapping steps. Using default parameters, we applied a workflow with three read classifiers (Centrifuge, KrakenUniq, Kraken2) and Re-mapping with Snippy, as available at <https://insaflu.insa.pt/>, to a set of simulated reads generated using ART [25] from emulated genome sequences with varying percentages of homogeneous nucleotide divergence from the SARS-CoV-2 reference genome (MN908947.3), ranging from 0.01% to 64%. A total of 1990 Illumina 150bp paired-end reads were artificial generated per sample, targeting a 10-fold depth of coverage. Both the artificial consensus sequences and reads are available at Zenodo repository (<https://zenodo.org/doi/10.5281/zenodo.10731592>).

As expected, a rise in genetic divergence resulted in a gradual reduction in the percentage of reads mapped (Fig. S**5A**). Still, even with a divergence as high as 32% (i.e., an artificial genome with 68% nucleotide identity to SARS-CoV-2), the few retained mapped reads (3%) would be sufficient to ensure a robust TELEVIR detection, as evidenced by the mapping metrics and features of the main report (e.g., 5 / 10 windows covered) (Fig. S**5B**). It is also noteworthy that all three read classifiers were able to detect genetic relatedness to SARS-CoV-2 in the sample even at higher divergence (40%), when Snippy no longer achieved successful mappings (using default parameters).

In summary, although the TELEVIR module is, by design, better suited for known pathogen’s diagnosis and surveillance rather than virus discovery, this simulation exercise opens good perspectives regarding its performance for detecting unknown viruses or very divergent variants. Indeed, in a real scenario, the percentage of divergence that would still allow mapping and identification is likely even higher than the percentage here identified, because mutations are accumulated heterogeneously across different regions of the genome, leading to some regions being more conserved that further potentiate classification and mapping of diverse variants.

**3.4.6 Reporting Statistics**

Coverage, the percent of genome coverage in bp, shows a weaker trend for Illumina, the relative difference between true and false positives being only 30 %. However, for ONT this difference is 50 % (Fig. S6 **A-B**).

The statistic “Windows Covered”, a summary of the distribution of mapped read across reference sequences, displays a markedly different distribution between true and false positive reference classifications: For Illumina, 25% and 75% true and false positive classifications have less than 80% of their genome covered, respectively; For ONT only 7 % of true positive classifications have less than 80 %, compared to 58 % of false positives (Fig. S6 **B-C**).

The proportion of reference sequences against which assembled contigs mapped successfully is 12 % if the references are false positives, 32 % for true positives. We find that this is largely due to influenza, SARS-CoV-2 and herpesvirus.

**4. Implementation**

**4.1 Software and Database selection**

In implementing the web and docker versions of the INSaFLU-TELEVIR pipeline, we kept several goals in mind: to provide a pipeline that is robust; to allow the user to run multiple pipelines as cross-validation; to take advantage of a modular pipeline tho give the user the flexibility to adjust to specific use-cases / constraints.

Our selection of the TELEVIR internals represents a trade-off of our analysis of the results of the benchmark with our understanding of what is considered to be adequate cross-validation, and was tempered by constraints in software implementation due to system requirements. At all steps, we strove to select those software / databases / parameter combinations indicated by our results to perform the best. However, considering that our benchmark does not cover all possible use cases, we also sought to keep read classification software that differed markedly in approach and substrate (DNA vs. AA).

As such, for the Viral Enrichment step, we implemented Centrifuge for both technologies. For the Assembly step, we implemented SPAdes and Raven for Illumina and ONT technologies, respectively. The assembler software Flye was tested in an early benchmark, but was dropped because of its low time performance. For the Contig Classification step, we implemented Blastn for both technologies. For the Read Classification step, we had to discard deSAMBA because of system requirements that are not met in the current INSaFLU-TELEVIR installation. The software Clark and Diamond frequently exceeded the memory limit in our machines and crashed, and were thus excluded as well. Finally, for ONT technologies we decided to keep four software: Centrifuge, FastViromeExplorer, Kaiju and KrakenUniq. This provides the user with two DNA based and two protein based algorithms. For Illumina technologies, we implemented Centrifuge, Kraken2 and KrakenUniq, two DNA and one protein based algorithm. Finally, for the Remapping step, we chose to keep Snippy for Illumina and Minimap2 for ONT.

**4.2 Final Report**

The TELEVIR Final Report was designed to provide an informative tool to investigate the presence of putative viral pathogens in biological samples, with a target audience to span all parties that deal with such data regardless of academic background. This sets a UX design challenge, which TELEVIR proposes to tackle by providing a rich report to cover most use cases. For this purpose we expanded the statistics extracted during the benchmark :

- Cov (%): horizontal coverage (i.e., percentage of the reference sequence covered)
- Depth: mean depth of coverage throughout the whole genome
- DepthC: mean depth of coverage exclusively in the covered regions
- Mapped reads: number of mapped reads
- start prop (%): number of mapped mapped reads divided by the number of input reads (after QC)
- mapped_prop (%): number of mapped reads divided by the number of reads used for mapping (i.e., reads retained after the “Virus enrichment” and/or “host depletion steps)
- Gaps: number of regions below the minimum coverage threshold (see note below)
- Windows Covered: proportion of windows with mapped reads. Reference sequences are split into windows (x), with window size and number (x) being a function of sequence length, from a minimum of 3 up to a maximum of 10. Window number (x) is calculated as the equal division of sequence length by 2000 (without remainder), i.e., sequences <8KB and >20KB result in 3 and 10 windows, respectively.
- class. success: indication of whether the TAXID was selected for mapping after reads and/or contigs classification
- mapping success: indication of whether reads/and contigs successfully mapped against the TAXID representative references sequence.

INSaFLU-TELEVIR documentation (latest) is provided at<http://insaflu.readthedocs.io/>.

**References**

1. Kim D, Song L, Breitwieser FP, Salzberg SL. Centrifuge: rapid and sensitive classification of metagenomic sequences. Genome Res [Internet]. 2016;26:1721–9. Available from: http://genome.cshlp.org/lookup/doi/10.1101/gr.210641.116

2. Ounit R, Wanamaker S, Close TJ, Lonardi S. CLARK: fast and accurate classification of metagenomic and genomic sequences using discriminative k-mers. BMC Genomics [Internet]. 2015;16:236. Available from: https://bmcgenomics.biomedcentral.com/articles/10.1186/s12864-015-1419-2

3. Wood DE, Lu J, Langmead B. Improved metagenomic analysis with Kraken 2. Genome Biol [Internet]. 2019;20:257. Available from: https://genomebiology.biomedcentral.com/articles/10.1186/s13059-019-1891-0

4. Wood DE, Salzberg SL. Kraken: ultrafast metagenomic sequence classification using exact alignments. Genome Biol [Internet]. 2014;15:R46. Available from: https://genomebiology.biomedcentral.com/articles/10.1186/gb-2014-15-3-r46

5. Menzel P, Ng KL, Krogh A. Fast and sensitive taxonomic classification for metagenomics with Kaiju. Nat Commun [Internet]. 2016;7:11257. Available from: https://www.nature.com/articles/ncomms11257

6. Li H, Durbin R. Fast and accurate short read alignment with Burrows-Wheeler transform. Bioinformatics [Internet]. 2009;25:1754–60. Available from: http://www.ncbi.nlm.nih.gov/pubmed/19451168

7. Henikoff S, Henikoff JG. Amino acid substitution matrices from protein blocks. Proc Natl Acad Sci [Internet]. 1992;89:10915–9. Available from: https://pnas.org/doi/full/10.1073/pnas.89.22.10915

8. Buchfink B, Reuter K, Drost H-G. Sensitive protein alignments at tree-of-life scale using DIAMOND. Nat Methods [Internet]. 2021;18:366–8. Available from: https://www.nature.com/articles/s41592-021-01101-x

9. Tithi SS, Aylward FO, Jensen R V, Zhang L. FastViromeExplorer: a pipeline for virus and phage identification and abundance profiling in metagenomics data. PeerJ. 2018;6:e4227.

10. Bray NL, Pimentel H, Melsted P, Pachter L. Near-optimal probabilistic RNA-seq quantification. Nat Biotechnol [Internet]. 2016;34:525–7. Available from: https://www.nature.com/articles/nbt.3519

11. Li G, Liu Y, Li D, Liu B, Li J, Hu Y, et al. Fast and Accurate Classification of Meta-Genomics Long Reads With deSAMBA. Front Cell Dev Biol [Internet]. 2021;9. Available from: https://www.frontiersin.org/articles/10.3389/fcell.2021.643645/full

12. Breitwieser FP, Baker DN, Salzberg SL. KrakenUniq: confident and fast metagenomics classification using unique k-mer counts. Genome Biol [Internet]. 2018;19:198. Available from: https://genomebiology.biomedcentral.com/articles/10.1186/s13059-018-1568-0

13. Altschul SF, Gish W, Miller W, Myers EW, Lipman DJ. Basic local alignment search tool. J Mol Biol [Internet]. 1990;215:403–10. Available from: https://linkinghub.elsevier.com/retrieve/pii/S0022283605803602

14. Li H. Minimap2: pairwise alignment for nucleotide sequences. Birol I, editor. Bioinformatics [Internet]. 2018;34:3094–100. Available from: https://academic.oup.com/bioinformatics/article/34/18/3094/4994778

15. Paez-Espino D, Chen I-MA, Palaniappan K, Ratner A, Chu K, Szeto E, et al. IMG/VR: a database of cultured and uncultured DNA Viruses and retroviruses. Nucleic Acids Res [Internet]. 2016;45. Available from: https://academic.oup.com/nar/article/doi/10.1093/nar/gkw1030/2333907

16. de Vries JJC, Brown JR, Fischer N, Sidorov IA, Morfopoulou S, Huang J, et al. Benchmark of thirteen bioinformatic pipelines for metagenomic virus diagnostics using datasets from clinical samples. J Clin Virol [Internet]. 2021;141:104908. Available from: https://linkinghub.elsevier.com/retrieve/pii/S138665322100175X

17. Carbo E, Sidorov I, van Rijn-Klink A, Pappas N, van Boheemen S, Mei H, et al. Performance of Five Metagenomic Classifiers for Virus Pathogen Detection Using Respiratory Samples from a Clinical Cohort. Pathogens [Internet]. 2022;11:340. Available from: https://www.mdpi.com/2076-0817/11/3/340

18. Bolger AM, Lohse M, Usadel B. Trimmomatic: a flexible trimmer for Illumina sequence data. Bioinformatics [Internet]. 2014;30:2114–20. Available from: https://academic.oup.com/bioinformatics/article/30/15/2114/2390096

19. Cantu VA, Sadural J, Edwards R. PRINSEQ++, a multi-threaded tool for fast and efficient quality control and preprocessing of sequencing datasets. PeerJ Prepr. 2019;7:e27553v1.

20. Ye SH, Siddle KJ, Park DJ, Sabeti PC. Benchmarking Metagenomics Tools for Taxonomic Classification. Cell [Internet]. 2019;178:779–94. Available from: https://linkinghub.elsevier.com/retrieve/pii/S0092867419307755

21. McIntyre ABR, Ounit R, Afshinnekoo E, Prill RJ, Hénaff E, Alexander N, et al. Comprehensive benchmarking and ensemble approaches for metagenomic classifiers. Genome Biol [Internet]. 2017;18:182. Available from: https://genomebiology.biomedcentral.com/articles/10.1186/s13059-017-1299-7

22. Meyer F, Bremges A, Belmann P, Janssen S, McHardy AC, Koslicki D. Assessing taxonomic metagenome profilers with OPAL. Genome Biol [Internet]. 2019;20:51. Available from: https://genomebiology.biomedcentral.com/articles/10.1186/s13059-019-1646-y

23. Sczyrba A, Hofmann P, Belmann P, Koslicki D, Janssen S, Dröge J, et al. Critical Assessment of Metagenome Interpretation—a benchmark of metagenomics software. Nat Methods [Internet]. 2017;14:1063–71. Available from: https://www.nature.com/articles/nmeth.4458

24. de Vries JJC, Brown JR, Couto N, et al. Recommendations for the introduction of metagenomic next-generation sequencing in clinical virology, part II: bioinformatic analysis and reporting. J Clin Virol. 2021;138:104812. doi:10.1016/j.jcv.2021.104812

25. Huang W, Li L, Myers JR, Marth GT. ART: a next-generation sequencing read simulator. Bioinformatics. 2012;28(4):593-594. doi:10.1093/bioinformatics/btr708
