# Supplementary material for: INSaFLU-TELEVIR: an open web-based bioinformatics suite for viral metagenomic detection and routine genomic surveillance
Source: Genome Med. 2024 Apr 25;16:61. doi: 10.1186/s13073-024-01334-3 (PMC11044337; doi:10.1186/s13073-024-01334-3)
Supplement: Supplementary file 1 — Additional file 1. Benchmark of the INSaFLU-TELEVIR pipeline for virus detection (TELEVIR): Resources, Workflow details, Benchmark and Implementation. Additional file 2. Benchmarking of INSaFLU against commonly used command line bioinformatics workflows for SARS-CoV-2 reference-based consensus generation (amplicon-based Illumina and ONT data), and validation of the INSaFLU snakemake pipeline. Additional file 3: Supplementary figures 1-8. Additional file 4: Supplementary tables 1-8. [file 13073_2024_1334_MOESM1_ESM.zip › Additional file 2.docx]

**Additional file 2**

Benchmarking of INSaFLU (<https://insaflu.insa.pt/>) against commonly used command line bioinformatics workflows for SARS-CoV-2 reference-based consensus generation (amplicon-based Illumina and ONT data), and validation of the INSaFLU snakemake pipeline (<https://github.com/INSaFLU/insaflu_snakemake>).

**Contents:**

**1. Illumina benchmark:** Goal, Datasets, Methods, Results

**2. ONT benchmark:** Goal, Datasets, Methods, Results

**3. INSaFLU Snakemake pipeline validation**

**4. Discussion**

**5. References**

1. **Illumina benchmark**

**Goal:** Comparison of SARS-CoV-2 consensus sequences generated with INSaFLU (<https://insaflu.insa.pt/>) with those obtained with one commonly used command line bioinformatics workflow (<https://github.com/andersen-lab/HCoV-19-Genomics>), involving BWA for reads mapping [1] and iVar (<https://github.com/andersen-lab/ivar>; <https://andersen-lab.github.io/ivar/html/manualpage.html>) for QC and consensus generation [2].

**Datasets:**

- *Dataset 1:* 154 SARS-CoV-2 Illumina paired-end samples from Baker et. al [3] available at the European Nucleotide Archive (ENA) under the BioProject PRJEB41737. Multiplex amplicons were prepared using the ARTIC nCoV-2019 V3 primer scheme (<https://github.com/artic-network/artic-ncov2019/tree/master/primer_schemes/nCoV-2019/>).

*- Dataset 2: 1000* SARS-CoV-2 Illumina paired-end samples from the Portuguese surveillance collection (<https://insaflu.insa.pt/covid19/>), collected late 2022, during Omicron BA.5 (BQ.1 and its sublineages) dominance (<https://insaflu.insa.pt/covid19/>), after human depletion with bmtagger (<https://ftp.ncbi.nlm.nih.gov/pub/agarwala/bmtagger/>) against the GRCh38 reference genome. Human-depleted reads are available at ENA under the BioProject PRJEB67829 Run IDs ERR12156465-ERR12157464). Multiplex amplicons were prepared using the ARTIC nCoV-2019 V4.1 primer scheme (<https://github.com/artic-network/artic-ncov2019/tree/master/primer_schemes/nCoV-2019/>), as previously described [4].

**Methods**

*- Software settings:*

- *INSaFLU:* online version 2.0.0 (<https://insaflu.insa.pt/>), used on 1 August 2023, using default parameters for QC filtering (Trimmomatic-0.39; SLIDINGWINDOW:5:20 LEADING:3 TRAILING:3 MINLEN:35 TOPHRED33), mutation calling (mutation frequency to assume in consensus >=51%) and consensus generation (namely, a minimum of 70% of the reference genome should be covered at least at 10-fold). The only exceptions from default parameters was masking the first 54bp and the last 67bp of the consensus for *Dataset 1* and masking the first 50bp and the last 76bp of the consensus for *Dataset 2*, corresponding to the first and last positions of the ARTIC nCoV-2019 V3 and V.4.1 primer schemes, respectively. Primer clipping to the respective ARTIC primer scheme was also turned ON, when applicable (as described below).
- *BWA/iVar pipeline* (<https://github.com/andersen-lab/ivar>; <https://andersen-lab.github.io/ivar/html/manualpage.html>) consulted on 1 August 2023, using default parameters, and adapting primer clipping to the respective ARTIC primer scheme.

*- Pipeline comparison and extra technical notes:*

For both pipelines, reference-based mapping, consensus generation and variant detection was performed against the Wuhan-Hu-1/2019 genome sequence (<https://www.ncbi.nlm.nih.gov/nuccore/MN908947>) [5]. To compare the results of both pipelines, we aligned the generated consensus using mafft (v7.520) [6]. When both sequences had more than 70% of the reference genome covered (current default for Illumina consensus generation in INSaFLU), we checked for nucleotide and indel differences between consensus of the same samples (excluding regions where at least one sequence had undefined nucleotides - Ns).

**Results:**

*- Dataset 1:*

A similar distribution of horizontal coverage (or N content) was observed between pipelines (Fig. S7A). 123/154 samples passed the default minimum coverage criteria for Illumina consensus generation in INSaFLU (i.e., a minimum of 70% of the reference genome covered at least at 10-fold). Applying the same 70% horizontal coverage cut-off (i.e., maximum 30% N content) to the consensus generated through the BWA/iVar pipeline, the same 123 samples pass this criteria. For 110/123 (89.4%) samples, no nucleotide (SNP or indels) differences were observed between the consensus reconstructed with both pipelines. Among the 13 samples having differences, 9 had 1 nucleotide difference, 3 had 2 differences, and 1 had 3 differences. A fine inspection revealed that a few differences fall within primer regions. As such, we extended the INSaFLU reference-based genome assembly pipeline by incorporating the iVar primer clipping functionality. Namely, we included the iVar primer trimming step (as described in iVar manual, but excluding the quality trimming, as this step is done upstream in INSaFLU pipeline), as well as the removal of reads containing minor variants matching primer sequence but not matching the overall consensus sequence. These steps are performed after read alignment, and before consensus generation, masking and variant calling, which is still performed as before, but taking as input the primer-clipped “bam” files. We ran again the updated INSaFLU pipeline over the Dataset 1, this time including the new step for primer trimming (ARTIC nCoV-2019 V3 primer scheme). In this case, 115/123 (93.5%) of the samples had no differences, 5 had 1 difference, 2 had 2 differences and 1 had 3 differences. A fine inspection of the 12 SNP/indel differences showed that they can be justified by two causes: i) 7 differences referred to mutations (SNPs or indels) with a frequency slightly below 50% (default INSaFLU minimum mutation frequency to assume in consensus; “minfrac” parameter), which were incorporated in the consensus by the BWA/iVAr pipeline; ii) 5 differences consisted of read mapping artifacts near deletions with coverage close to 10x. Of note, the latter differences are not observed if a stricter 30x depth of coverage threshold is applied (as performed in the Portuguese SARS-CoV-2 routine surveillance).

*- Dataset 2:*

Following the results of the Dataset 1 (including samples from an early stage of the pandemic and thus, with a low average of ~13 mutations to the Wuhan-1 reference), we sought to explore the pipelines’ congruence with a more recent dataset containing a considerably higher number of mutations (~80 mutations per sample) to be called and incorporated in the consensus. For that, we selected 1000 Illumina paired-end samples from the Portuguese surveillance collection (<https://insaflu.insa.pt/covid19/>), collected late 2022. In this comparison, the updated INSaFLU pipeline (with primer clipping) was applied. A similar distribution of percentage horizontal coverage (or N content) was observed between pipelines (Fig. S7B). 933/1000 samples passed the default minimum coverage criteria for consensus generation in INSaFLU (i.e., a minimum of 70% of the reference genome covered at least at 10-fold). Applying the same 70% horizontal coverage cut-off (i.e., maximum 30% N content) to the consensus generated through the BWA/iVar pipeline, the same 933 samples pass this criteria. For 900/933 (96.4%%) samples, no nucleotide (SNP or indels) differences were observed between the consensus reconstructed with both pipelines. In total, we observed 35 differences across more than 70000 total mutations detected in all samples. A fine inspection of the 33 samples with discrepancies (31 with 1 nucleotide difference, and 2 with 2 differences) revealed that all were mutations close to the default threshold of 50%, with most of the cases also being in regions of very low coverage (below the 30x used for the Portuguese SARS-CoV-2 routine surveillance).

1. **Oxford Nanopore Technologies (ONT)**

**Goal:** Comparison of SARS-CoV-2 consensus sequences generated with INSaFLU (<https://insaflu.insa.pt/>) with those obtained with one commonly used command line bioinformatics workflow for ONT data, the Artic Network pipeline (<https://github.com/artic-network/fieldbioinformatics>).

**Datasets:**

*- Dataset 1:* 297 SARS-CoV-2 ONT reads from Baker et. al [3] available at the ENA under the BioProject PRJEB41737. Multiplex amplicons were prepared using the ARTIC nCoV-2019 V3 primer scheme (<https://github.com/artic-network/artic-ncov2019/tree/master/primer_schemes/nCoV-2019/>). This dataset is part of a set of CDC benchmark datasets for WGS analysis of SARS-CoV-2 (<https://github.com/CDCgov/datasets-sars-cov-2>).

*- Dataset 2:* 153 SARS-CoV-2 ONT reads from Bull et al., 2020 [7] available at the ENA under the BioProject PRJNA675364. Of note, 4 out the 157 Bioproject samples had larger fastq files than what the public INSaFLU website allows, and were discarded from the comparison. Multiplex amplicons were prepared using the ARTIC nCoV-2019 V3 primer scheme (<https://github.com/artic-network/artic-ncov2019/tree/master/primer_schemes/nCoV-2019/>).

**Methods**

*- Software settings:*

- *INSaFLU:* online version 2.0.0 (<https://insaflu.insa.pt/>), used on 1 August 2023, using default parameters for QC filtering (NanoFilt-2.6.0; -q 8 -l 50 --headcrop 30 --tailcrop 30 --maxlength 50000), mutation validation (mutation frequency to assume in consensus >80%, with positions with mutations with frequency between 50% and 80% being masked with ‘N’) and consensus generation (namely, a minimum of 89% of the reference genome should be covered at least at 30x). The only exceptions from default parameters were: i) masking the first 54bp and the last 67bp of the consensus, corresponding to the first and last positions of the ARTIC nCoV-2019 V3 schemes, respectively; ii) turned ON ARTIC nCoV-2019 V3 scheme primer clipping, when applicable (using iVar as implemented for Illumina).
- ARTIC Network pipeline (<https://github.com/artic-network/fieldbioinformatics>), consulted on 1 August 2023 (artic version 1.2.3), was run using the “medaka” mode (as no fast5 were available), model r941_min_high_g360, with default parameters, and setting the ARTIC nCoV-2019 V3 primer scheme (<https://github.com/artic-network/artic-ncov2019/tree/master/primer_schemes/nCoV-2019/>).

*- Pipeline comparison and extra technical notes:*

For both pipelines, reference-based mapping, consensus generation and variant detection was performed against the Wuhan-Hu-1/2019 genome sequence (<https://www.ncbi.nlm.nih.gov/nuccore/MN908947>) [5]. To compare the results of both pipelines, we aligned the generated consensus using mafft (v7.520) [6]. When both sequences had more than 90% of the reference genome covered (current default for Illumina consensus generation in INSaFLU), we checked for nucleotide and indel differences between consensus of the same samples (excluding regions where at least one sequence had undefined nucleotides - Ns).

**Results:**

*- Dataset 1:*

A similar distribution of horizontal coverage (or N content) was observed between pipelines (Fig. S8A). 204/297 samples passed the default minimum coverage criteria for consensus generation in INSaFLU (i.e., ≥90% of the reference genome covered at least at 30x). Applying the same coverage cut-off to the consensus generated through the ARTIC pipeline, 203/297 samples pass this criteria, with 201 being common for both pipelines. For 171/201 (85.1%) samples, no nucleotide (SNP or indels) differences were observed between the consensus reconstructed with both pipelines. Among the 30 samples having differences, 26 had 1 difference, 3 had 2 differences and 1 had 3 differences. A fine inspection of the 35 SNP/indel differences showed that 32 were positions with mixed nucleotide composition overlapping primer regions. Thus, similarly to illumina, we also introduced the possibility of performing primer trimming on ONT data. When doing this with the samples of this dataset, 197/297 samples passed the default ONT coverage criteria. Of the consensus generated using the INSaFLU ONT pipeline with iVar clipping, 191/197 (97%) had no differences when comparing with the ARTIC generated consensus sequences.

*- Dataset 2:*

In order to complement the results for Dataset 1, we tested the upgraded ONT INSaFLU pipeline (with iVar primer clipping) another publicly available dataset from Bull et al., 2020 [7]. 153/153 samples passed the default minimum coverage criteria for consensus generation in INSaFLU (i.e., ≥90% of the reference genome covered at least at 30x). Applying the same coverage cut-off to the consensus generated through the ARTIC pipeline, only 108/153 samples passed this criteria, including 20 for which the ARTIC pipeline failed. In line with this observation, the distribution of horizontal coverage (or N content) was also slightly different, showing that the ARTIC pipeline tended to insert more Ns, at least for this Dataset (Fig. S8A). For 100/108 (92.6%) samples that could be compared, no nucleotide (SNP or indels) differences were observed between the consensus reconstructed with both pipelines. All of the differences are due to insertion/deletion misalignments occurring in a homopolymeric- and T-rich region (11025-11125) of the SARS-CoV-2 Wuhan-1 reference genome.

1. **INSaFLU snakemake pipeline validation**

A snakemake pipeline to run the surveillance-oriented reference-based genome assembly INSaFLU component (for both ONT and Illumina) was developed and released at <https://github.com/INSaFLU/insaflu_snakemake>. To validate it, we have compared the consensus obtained from the INSaFLU website with the consensus obtained from the snakemake pipeline, running with the same parameters on the same datasets as reported above. The INSaFLU snakemake workflow produced the same consensus sequences as the public website.

1. **Discussion:**

Here, we performed a benchmarking for SARS-CoV-2 reference-based consensus generation, comparing INSaFLU with commonly used command-line bioinformatics workflows for Illumina and ONT data, using two independent datasets per sequencing technology.

For Illumina, we started by performing an initial assessment with a dataset (here called Dataset 1) described in Baker et. al. [3], and suggested for benchmarking WGS analysis of SARS-CoV-2 by CDC (<https://github.com/CDCgov/datasets-sars-cov-2>). Despite the considerably high congruence in consensus sequences generated by the two pipelines (INSaFLU versus BWA/iVar), these initial results underlined the expected added value of incorporating an extra step of targeted primer clipping from the BAM file into the INSaFLU pipeline, which was implemented in the INSaFLU platform (<https://insaflu.insa.pt/>). When running the upgraded INSaFLU pipeline over a larger and more diverse Dataset (Dataset 2), the congruence between the pipelines was highly satisfactory, with 96.4% of the compared samples having no nucleotide (SNP or indels) differences and a total of just 35 SNP/indel differences being observed across the more than 70000 mutations called across all samples. All these differences could be justified by pipeline differences in frequency criteria to assume a mutation in consensus (>=51% in INSaFLU and the “majority base” in BWA/iVar) or by mutation calling in low coverage regions (close to 10-fold). This low 10x cut-off was used in this benchmark exercise to reflect the current default value in the INSaFLU general settings, but we routinely use a threshold of 30x for the Portuguese SARS-CoV-2 routine surveillance. As such, these benchmark results also corroborate our choice for a stricter threshold of 30x, which we hereby recommend when using INSaFLU for SARS-CoV-2 reference-based consensus reconstruction. Of note, we should highlight that the INSaFLU settings applied during the genomic surveillance of SARS-CoV-2 in Portugal before the incorporation of the iVar primer clipping step [4,8,9] included an extra Trimmomatic step to cut 30 bases from the start of all reads (HEADCROP=30), another strategy to minimize primer-related issues.

For ONT, a similar scenario was obtained through the comparison of INSaFLU ONT pipeline with the broadly used ARTIC pipeline. Indeed, a satisfactory consensus sequence congruence was achieved, with the few differences observed falling in regions typically challenging for ONT sequencing, namely homopolymeric tracts. We should highlight that, while the applied ARTIC pipeline (via medaka “mode”) lacks a QC step before mapping (contrarily to the INSaFLU workflow, which relies on NanoFilt), INSaFLU cannot handle fast5 files. As such, this benchmark exercise did not assess the performance of the ARTIC pipeline using signal data (via “[nanopolish](https://github.com/jts/nanopolish)” mode), which might justify the observed over-incorporation of undefined nucleotides (N) by the ARTIC (medaka) pipeline, specially in Dataset 2.

Finally, this study also demonstrated the full congruence between the newly developed INSaFLU command-line snakemake pipeline (<https://github.com/INSaFLU/insaflu_snakemake>) and the public website ((<https://insaflu.insa.pt/>) for consensus generation, thus expanding the potential usage of both ONT and Illumina INSaFLU pipelines in other contexts (e.g., compute clusters).

1. **References**

1. Li H, Durbin R. Fast and accurate short read alignment with Burrows-Wheeler transform. Bioinformatics [Internet]. 2009;25:1754–60. Available from: http://www.ncbi.nlm.nih.gov/pubmed/19451168

2. Grubaugh ND, Gangavarapu K, Quick J, Matteson NL, De Jesus JG, Main BJ, et al. An amplicon-based sequencing framework for accurately measuring intrahost virus diversity using PrimalSeq and iVar. Genome Biol [Internet]. 2019;20:8. Available from: http://www.ncbi.nlm.nih.gov/pubmed/30621750

3. Baker DJ, Aydin A, Le-Viet T, Kay GL, Rudder S, de Oliveira Martins L, et al. CoronaHiT: high-throughput sequencing of SARS-CoV-2 genomes. Genome Med [Internet]. 2021;13:21. Available from: https://genomemedicine.biomedcentral.com/articles/10.1186/s13073-021-00839-5

4. Borges V, Isidro J, Cortes-Martins H, Duarte S, Vieira L, Leite R, et al. Massive dissemination of a SARS-CoV-2 Spike Y839 variant in Portugal. Emerg Microbes Infect [Internet]. 2020;9:2488–96. Available from: https://www.tandfonline.com/doi/full/10.1080/22221751.2020.1844552

5. Wu F, Zhao S, Yu B, Chen Y-M, Wang W, Song Z-G, et al. Author Correction: A new coronavirus associated with human respiratory disease in China. Nature [Internet]. 2020;580:E7–E7. Available from: https://www.nature.com/articles/s41586-020-2202-3

6. Katoh K, Standley DM. MAFFT Multiple Sequence Alignment Software Version 7: Improvements in Performance and Usability. Mol Biol Evol [Internet]. 2013;30:772–80. Available from: https://academic.oup.com/mbe/article-lookup/doi/10.1093/molbev/mst010

7. Bull RA, Adikari TN, Ferguson JM, Hammond JM, Stevanovski I, Beukers AG, et al. Analytical validity of nanopore sequencing for rapid SARS-CoV-2 genome analysis. Nat Commun [Internet]. 2020;11:6272. Available from: https://www.nature.com/articles/s41467-020-20075-6

8. Borges V, Isidro J, Macedo F, Neves J, Silva L, Paiva M, et al. Nosocomial Outbreak of SARS-CoV-2 in a “Non-COVID-19” Hospital Ward: Virus Genome Sequencing as a Key Tool to Understand Cryptic Transmission. Viruses [Internet]. 2021;13:604. Available from: https://www.mdpi.com/1999-4915/13/4/604

9. Borges V, Isidro J, Trovão NS, Duarte S, Cortes-Martins H, Martiniano H, et al. SARS-CoV-2 introductions and early dynamics of the epidemic in Portugal. Commun Med [Internet]. 2022;2:10. Available from: https://www.nature.com/articles/s43856-022-00072-0
