# Supplementary material for: INSaFLU-TELEVIR: an open web-based bioinformatics suite for viral metagenomic detection and routine genomic surveillance
Source: Genome Med. 2024 Apr 25;16:61. doi: 10.1186/s13073-024-01334-3 (PMC11044337; doi:10.1186/s13073-024-01334-3)
Supplement: Supplementary file 1 — Additional file 1. Benchmark of the INSaFLU-TELEVIR pipeline for virus detection (TELEVIR): Resources, Workflow details, Benchmark and Implementation. Additional file 2. Benchmarking of INSaFLU against commonly used command line bioinformatics workflows for SARS-CoV-2 reference-based consensus generation (amplicon-based Illumina and ONT data), and validation of the INSaFLU snakemake pipeline. Additional file 3: Supplementary figures 1-8. Additional file 4: Supplementary tables 1-8. [file 13073_2024_1334_MOESM1_ESM.zip › Supplementary_Figures_1_to_8.pdf]

# Supplementary Figures

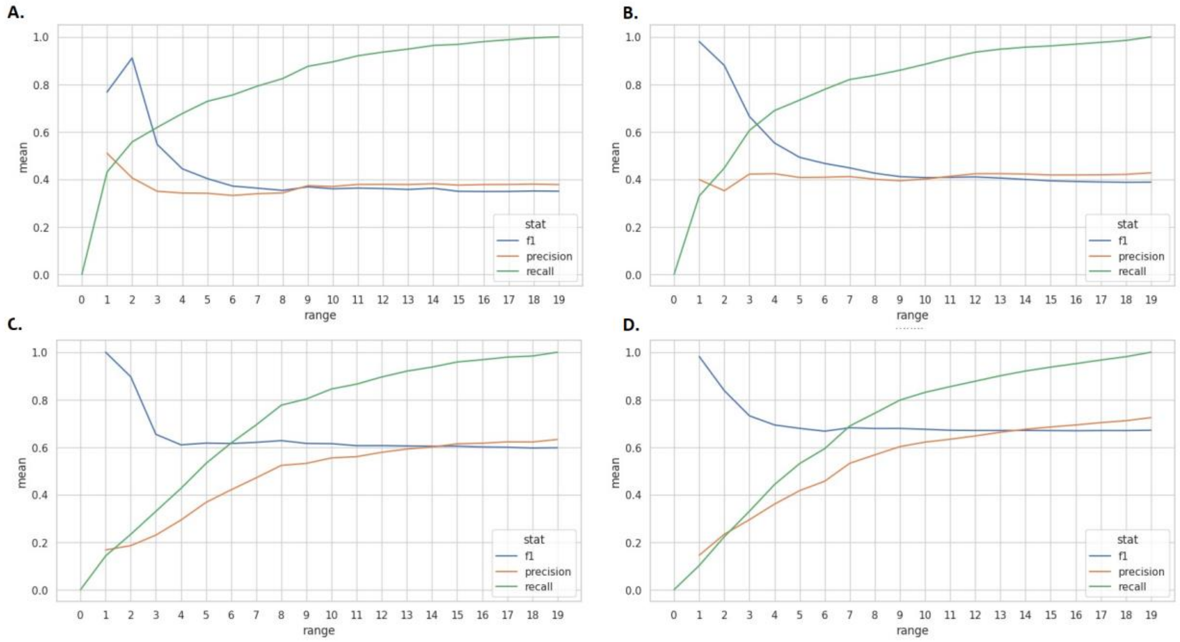


**Fig. S1**. Classification performance estimates of the final report size and sorting algorithm, as measured relative to intermediate classification output - see methods. The final report precision, recall and f1 statistics (y-axis) are plotted as a function of final report sample size (x-axis), with simple sort and combined sort (top vs bottom respectively), for ONT and Illumina Benchmarks (left vs right respectively). **A** - Simple sort ONT; **B** - Simple Sort Illumina; **C** - Combined Sort ONT; **D** - Combined Sort - Illumina.

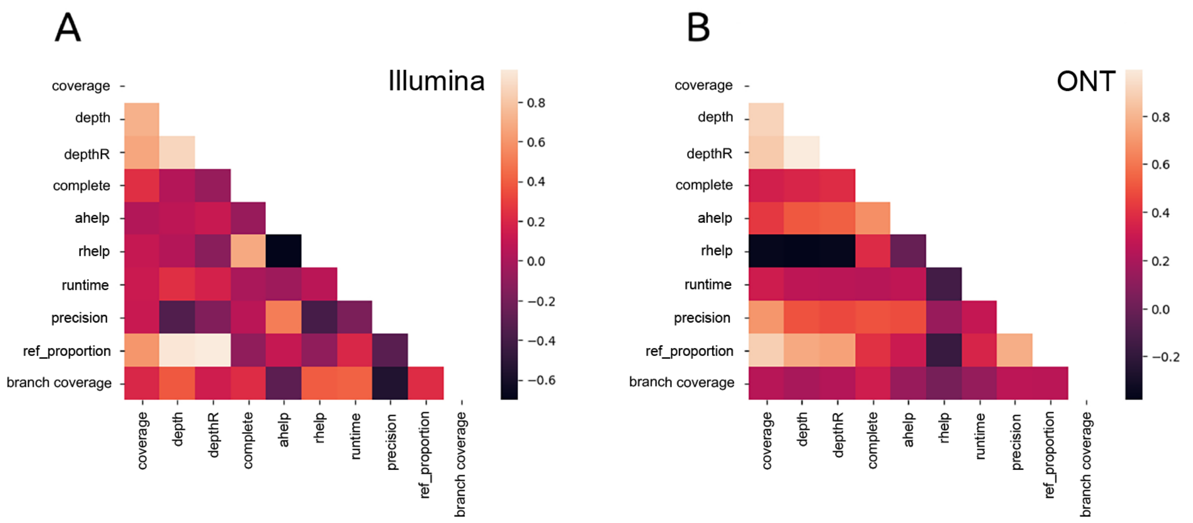


**Fig. S2**. Correlation heatmap between benchmark statistics across workflows. Statistics are averaged across samples for each workflow (A – Illumina; B – ONT). Runtime, ref_proportion, depth, depthR and coverage were standardized by division by maxima by sample. ahelp: proportion of references identified through contig classification; rhelp: proportion of references identified through reads classification.

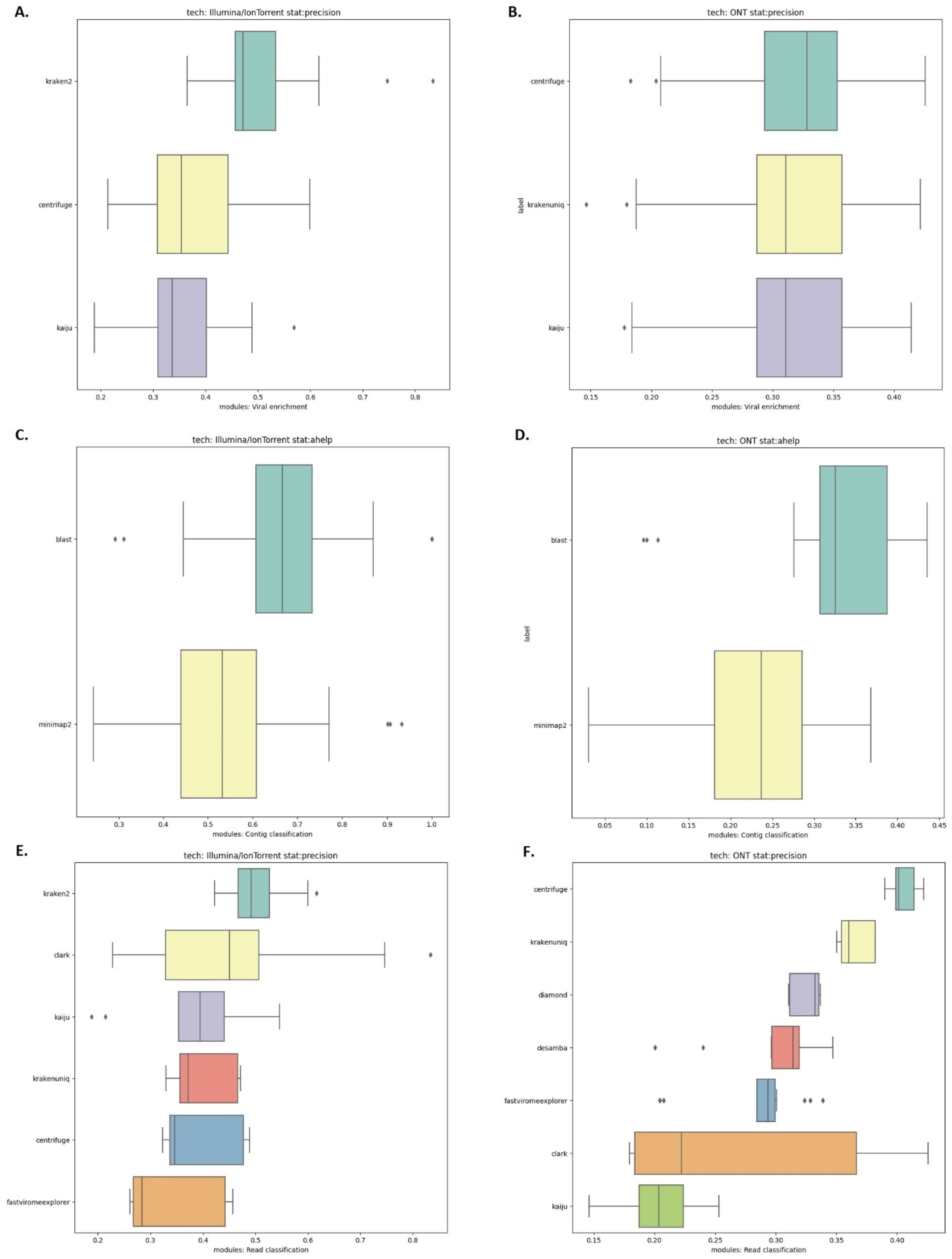


**Fig. S3**. Comparison of TELEViR Software by module. Left: Illumina; right: ONT; Top (**A-B**) - Viral enrichment, precision; Middle (**C-D**): Contig Classification, proportion of true positive accessions identified using contigs; Bottom (**E-F**) - Read Classification, precision.

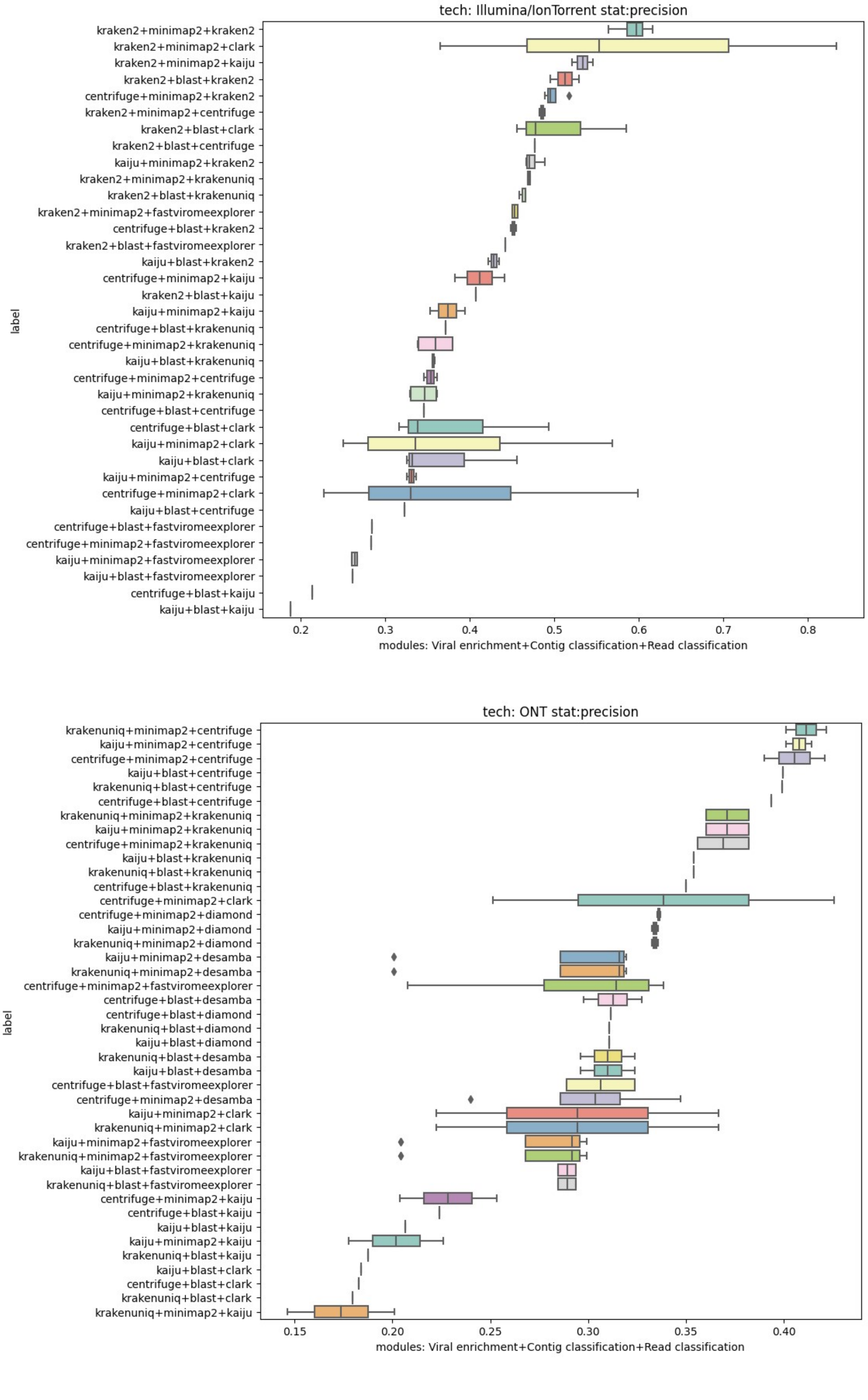


**Fig. S4**. Precision by TELEVIR metagenomics pipeline, sorted in decreasing order.

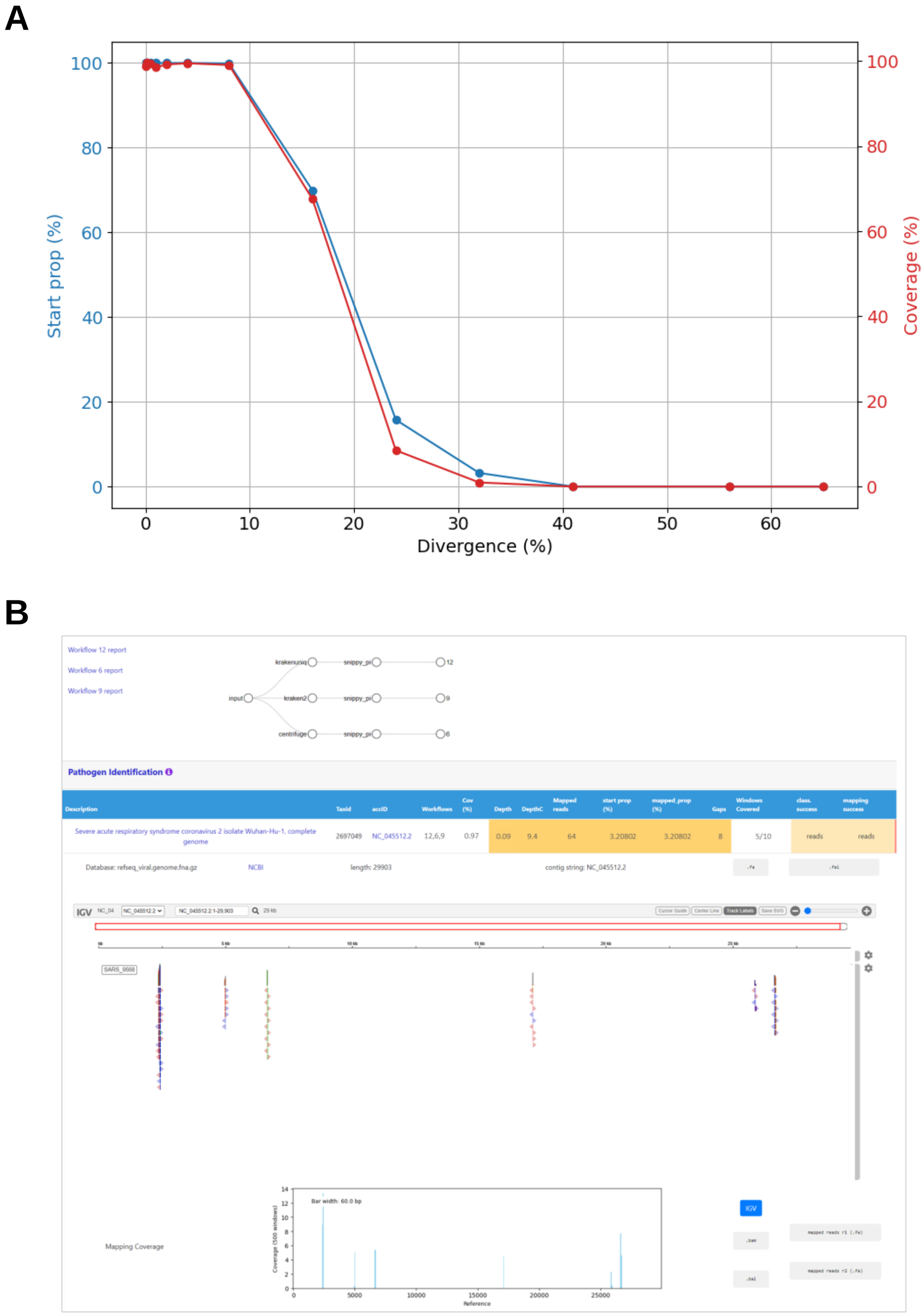


**Fig. S5. A.** Simulation of the impact of incremental nucleotide divergence on the mapping performance, as assessed by the number of mapped reads divided by the number of input reads [Start prop (%)] and the percentage of the reference sequence covered [Coverage (%)]. **B.** Screenshot of TELEVIR mapping report for the simulated sample with 32% divergence to SARS-CoV-2, showing the detection of SARS-CoV-2 as the first hit and the mapped reads dispersed across genome (which is a intuitive metagenomics indicator of a true positive hit)

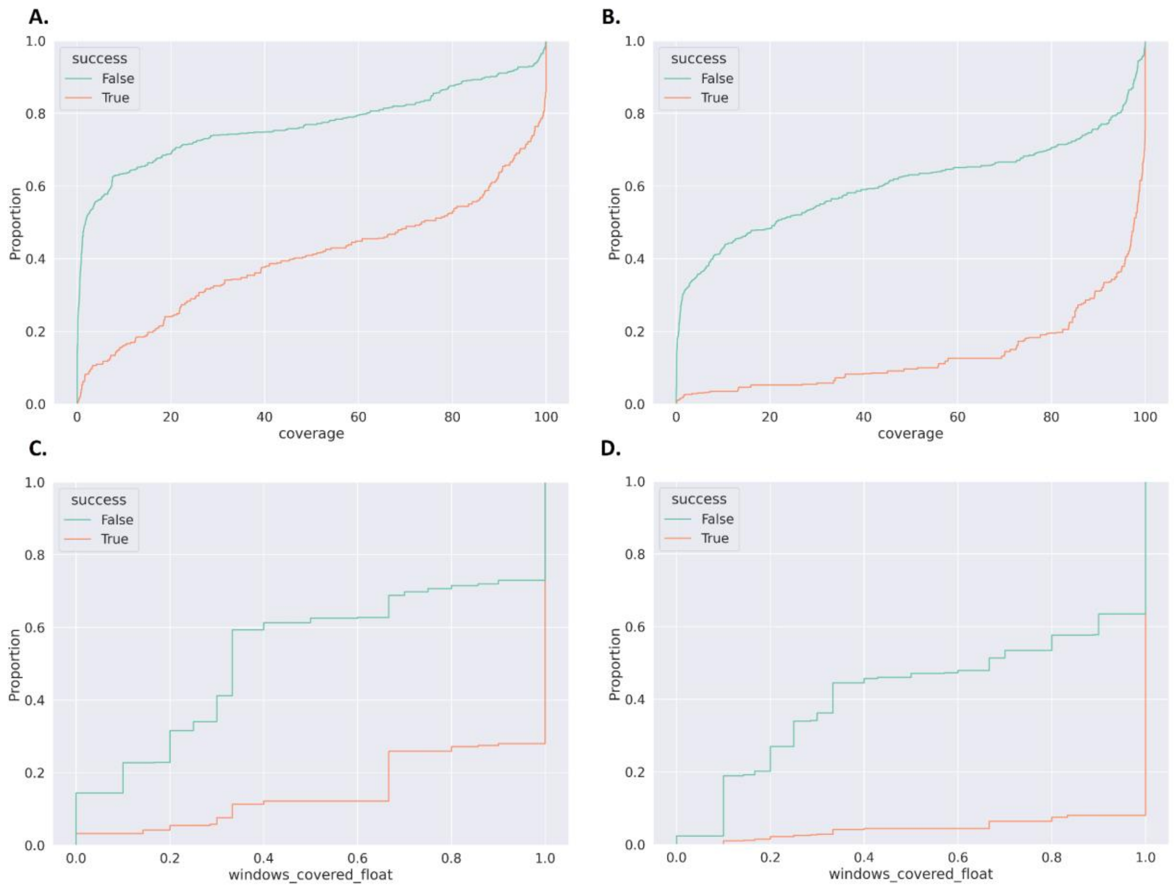


**Fig. S6.** Reporting statistics for True and False positives. **A-B**: Coverage cumulative distribution for True and False positive classifications, for Illumina and ONT reads respectively. **C-D**: Windows covered, as a proportion, cumulative distribution for True and False positive classifications, for Illumina and ONT reads, respectively.

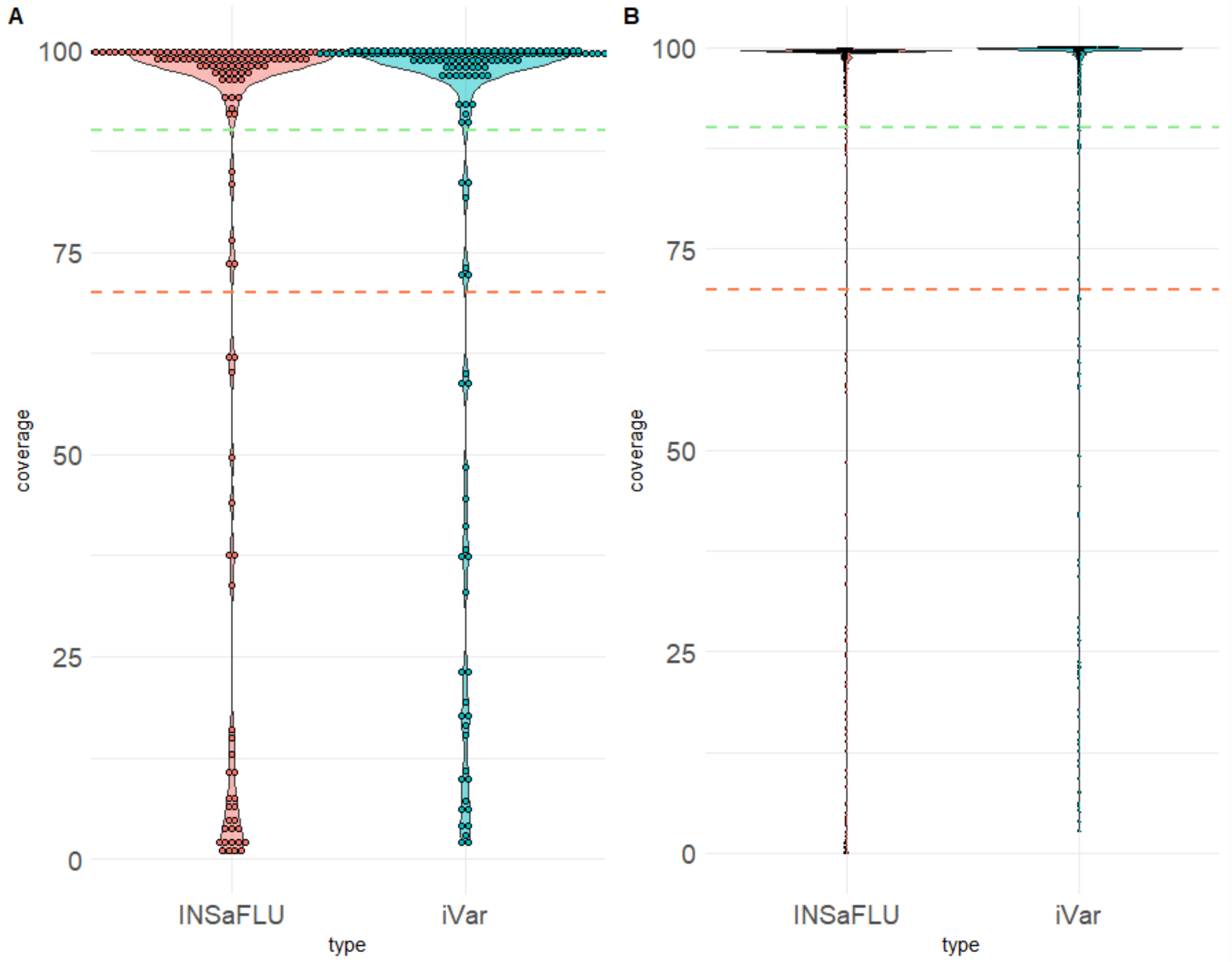


**Fig. S7.** INSaFLU Illumina benchmarking - sample horizontal coverage (%) of samples from INSaFLU and BWA/iVar. A) Dataset 1. B) Dataset 2.

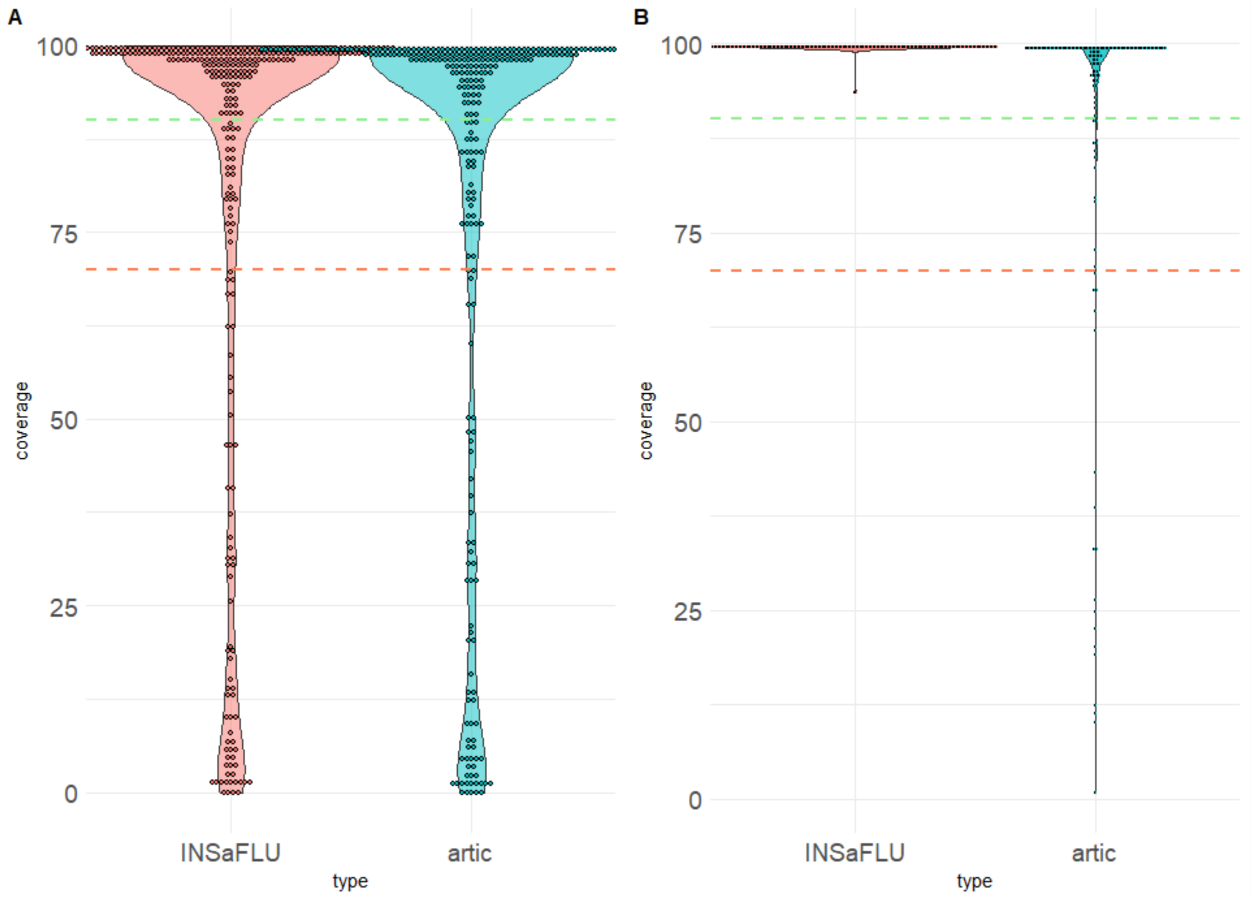


**Fig. S8.** INSaFLU ONT benchmarking - Sample horizontal coverage (%) of samples from INSaFLU and Artic pipeline. A) Dataset 1. B) Dataset 2. In the case of B, INSaFLU was run with primer clipping. Moreover, the plot for artic does not include 20 samples for which no results could be generated.
